# ECHDC1 knockout mice accumulate ethyl-branched lipids and excrete abnormal intermediates of branched-chain fatty acid metabolism

**DOI:** 10.1016/j.jbc.2021.101083

**Published:** 2021-08-19

**Authors:** Joseph P. Dewulf, Stéphanie Paquay, Etienne Marbaix, Younès Achouri, Emile Van Schaftingen, Guido T. Bommer

**Affiliations:** 1 Department of Biochemistry, de Duve Institute, UCLouvain, Brussels, Belgium; 2 Walloon Excellence in Lifesciences and Biotechnology (WELBIO), Brussels, Belgium; 3 Department of Laboratory Medicine, University Hospital St Luc, UCLouvain, Bruxelles, Belgium; 4 Department of Neuropediatrics, University Hospital St Luc, UCLouvain, Bruxelles, Belgium; 5 Department of Anatomical Pathology, University Hospital St Luc, UCLouvain, Bruxelles, Belgium; 6 Department of Cell Biology, de Duve Institute, UCLouvain, Bruxelles, Belgium; 7 Transgenesis Platform, de Duve Institute, UCLouvain, Bruxelles, Belgium

**Keywords:** ECHDC1, branched-chain FAs, methyl-branched fatty acids, ethyl-branched fatty acids, ethylmalonyl-CoA, 2,2-dimethylmalonyl-CoA, 2,2-dimethylmalonic acid, ethylmalonic acid, acylglycine, acyltaurine, plasmanylcholine, ACC, acetyl-CoA carboxylase, BAT, brown adipose tissue, CDI, carbonyldiimidazole, ECHDC1, enzyme ethylmalonyl-CoA decarboxylase, em-CoA, ethylmalonyl-CoA, FA, fatty acid, FASN, fatty acid synthase, mm-CoA, methylmalonyl-CoA, SIM, selected-ion monitoring

## Abstract

The cytosolic enzyme ethylmalonyl-CoA decarboxylase (ECHDC1) decarboxylates ethyl- or methyl-malonyl-CoA, two side products of acetyl-CoA carboxylase. These CoA derivatives can be used to synthesize a subset of branched-chain fatty acids (FAs). We previously found that ECHDC1 limits the synthesis of these abnormal FAs in cell lines, but its effects *in vivo* are unknown. To further evaluate the effects of ECHDC1 deficiency, we generated knockout mice. These mice were viable, fertile, showed normal postnatal growth, and lacked obvious macroscopic and histologic changes. Surprisingly, tissues from wild-type mice already contained methyl-branched FAs due to methylmalonyl-CoA incorporation, but these FAs were only increased in the intraorbital glands of ECHDC1 knockout mice. In contrast, ECHDC1 knockout mice accumulated 16–20-carbon FAs carrying ethyl-branches in all tissues, which were undetectable in wild-type mice. Ethyl-branched FAs were incorporated into different lipids, including acylcarnitines, phosphatidylcholines, plasmanylcholines, and triglycerides. Interestingly, we found a variety of unusual glycine-conjugates in the urine of knockout mice, which included adducts of ethyl-branched compounds in different stages of oxidation. This suggests that the excretion of potentially toxic intermediates of branched-chain FA metabolism might prevent a more dramatic phenotype in these mice. Curiously, ECHDC1 knockout mice also accumulated 2,2-dimethylmalonyl-CoA. This indicates that the broad specificity of ECHDC1 might help eliminate a variety of potentially dangerous branched-chain dicarboxylyl-CoAs. We conclude that ECHDC1 prevents the formation of ethyl-branched FAs and that urinary excretion of glycine-conjugates allows mice to eliminate potentially deleterious intermediates of branched-chain FA metabolism.

Most fatty acids (FAs) have a linear carbon backbone. Their synthesis starts with acetyl-CoA and proceeds by repeated incorporation of two carbon units from malonyl-CoA to generate a linear carbon chain. Branched FAs are rare in mammals. These compounds derive from food intake, the intestinal microbiota, or *de novo* synthesis. They can be derived from isoprenoid compounds, but they can also be synthesized from branched precursors due to the promiscuity of fatty acid synthase (FASN) (1). On the one hand, acetyl-CoA can be replaced by branched-chain amino acid breakdown products, leading to the formation of a FA with a methyl-branch on the penultimate (“*iso*-FA“) or the antepenultimate carbon (“*anteiso-*FA”) (1). On the other hand, but less efficiently, FASN is able to incorporate methylmalonyl-CoA (mm-CoA) or ethylmalonyl-CoA (em-CoA) unit(s) instead of malonyl-CoA, producing methyl or ethyl branches on even-numbered carbon atoms (Fig. 1*A*) (2, 3).

**Figure 1:**
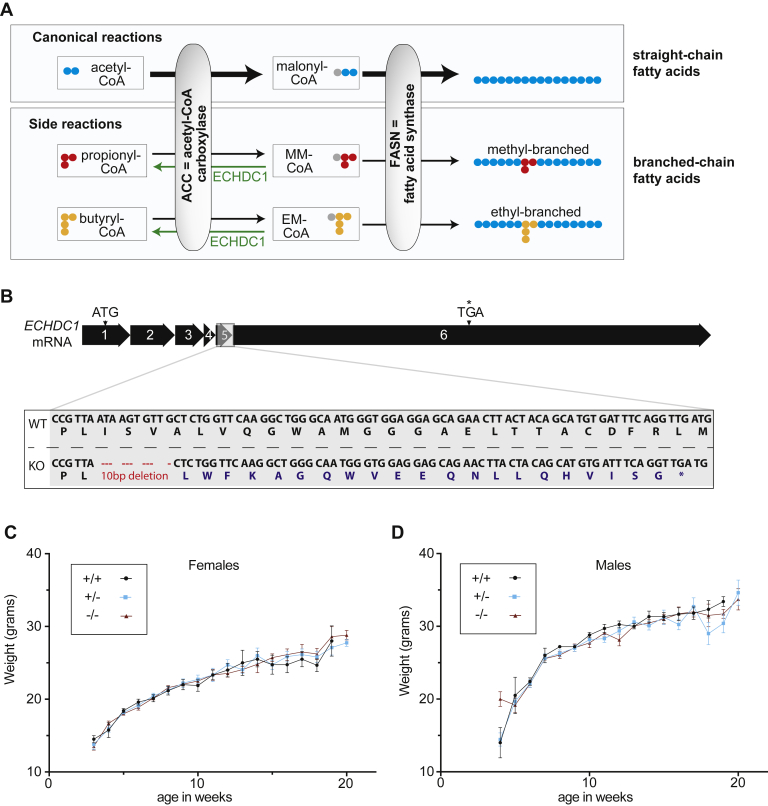
**ECHDC1 function and mouse model.***A*, ECHDC1 is a metabolite repair enzyme, mainly found in the cytosol, which decarboxylates side products of acetyl-CoA carboxylase (ACC), namely ethylmalonyl-CoA and methylmalonyl-CoA. Although less efficiently used than malonyl-CoA, these metabolites can serve as substrate for fatty acid synthase (FASN), resulting in the synthesis of branched-chain fatty acids. *B*, ECHDC1 exons, mRNA, and corresponding translation in *Mus musculus.* The position of the 10-bp deletion generated in the KO mouse model and the resulting frameshift and premature stop codon (∗) are highlighted in *gray*. *C*, weight progress curves in female WT controls (n = 9), heterozygous controls (n = 27) and homozygous KO (n = 26) mice. *D*, weight progress curves in male WT controls (n = 7), heterozygous controls (n =26), and homozygous KO (n = 28) mice. ECHDC1, ethylmalonyl-CoA decarboxylase; EM-CoA, ethylmalonyl-CoA; MM-CoA, methylmalonyl-CoA.

Incorporation of branched-chain FAs can affect membrane fluidity (4). The abundance of some *iso*- and *anteiso*-branched-chain FAs is inversely correlated with vitamin B12 levels and potentially with obesity (1, 5). In contrast, very little is known about the abundance and function of branched-chain FAs, where the branches are not occurring close to the end of the carbon chain.

ECHDC1 (Enoyl-CoA Hydratase Domain-Containing Protein 1) is located mainly in the cytosol and decarboxylates em-CoA and to a lesser extent, mm-CoA (6). While mm-CoA is a physiological metabolite in mitochondria, both mm-CoA and em-CoA can also be formed in the cytosol when acetyl-CoA carboxylase (ACC) erroneously carboxylates propionyl-CoA and butyryl-CoA, respectively (7) (Fig. 1*A*). Their decarboxylation by ECHDC1 in the cytosol prevents them from being used in FA synthesis and is therefore expected to prevent branched-chain FA synthesis (Fig. 1*A*). Consistent with this, we recently showed that in the absence of ECHDC1, methyl-branched FAs strongly increase in mouse adipocyte cell models (3T3-L1 and L929). In addition, supplementation of isotope-labeled ethylmalonate revealed that low levels of ethyl-branched FAs were also synthesized in ECHDC1 KO cells (2).

Lipid metabolism in a living organism is complicated by the collaboration between different organs. Accumulation of ethyl-branched FAs might be expected to lead to toxic effects for at least three reasons: (1) Their degradation in canonical β-oxidation is likely limited (8). (2) Branched-chain FAs could perturb membranes by getting incorporated in phospholipids and thereby affect the function of a wide range of organs. (3) Several peroxisomal disorders due to the accumulation of branched-chain FAs are known to cause severe neurological diseases due to the accumulation of pristanic and/or phytanic derivatives, two isoprenoid-derived polymethylated branched FAs (9, 10). We decided to generate an ECHDC1-deficient mouse model to determine whether and which branched-chain FAs were accumulating. We observed strong increases in ethyl-branched-chain FAs that were incorporated into a wide range of complex lipids. Surprisingly, knockout mice were phenotypically normal, potentially because the excretion of a large number of products of abortive FA synthesis in the urine prevented excessive accumulation of branched-chain FAs.

## Results

### ECHDC1 knockout mice are viable and do not show any obvious health defects

To investigate the role of ECHDC1 *in vivo*, we used the CRISPR/Cas9 genome-editing system to generate a mouse line with a 10 bp deletion in exon 5 of the ECHDC1 gene (Fig. 1*B*). This led to a frameshift and a premature stop codon before key catalytic sites of ECHDC1. ECHDC1−/− mice were born at the frequency expected from a Mendelian inheritance of the mutated allele. They did not show any gross abnormalities and showed postnatal growth that was indistinguishable from that of sibling controls (Fig. 1*C*). Microscopic analysis of hematoxylin-eosin (HE) stained sections of different tissues (see Experimental Procedures) obtained from mice aged between 4 and 10 months did not reveal reproducible anomalies. Furthermore, frozen sections of the liver, kidney, heart, as well as of white and BAT from 9-month-old mice were stained with Oil Red O to compare the accumulation of neutral lipids, but no significant difference was found between heterozygous and knockout mice.

### Ethyl-branched fatty acids are found only in ECHDC1 knockout mouse tissues, especially in brown adipose tissue (BAT)

**Figure 2:**
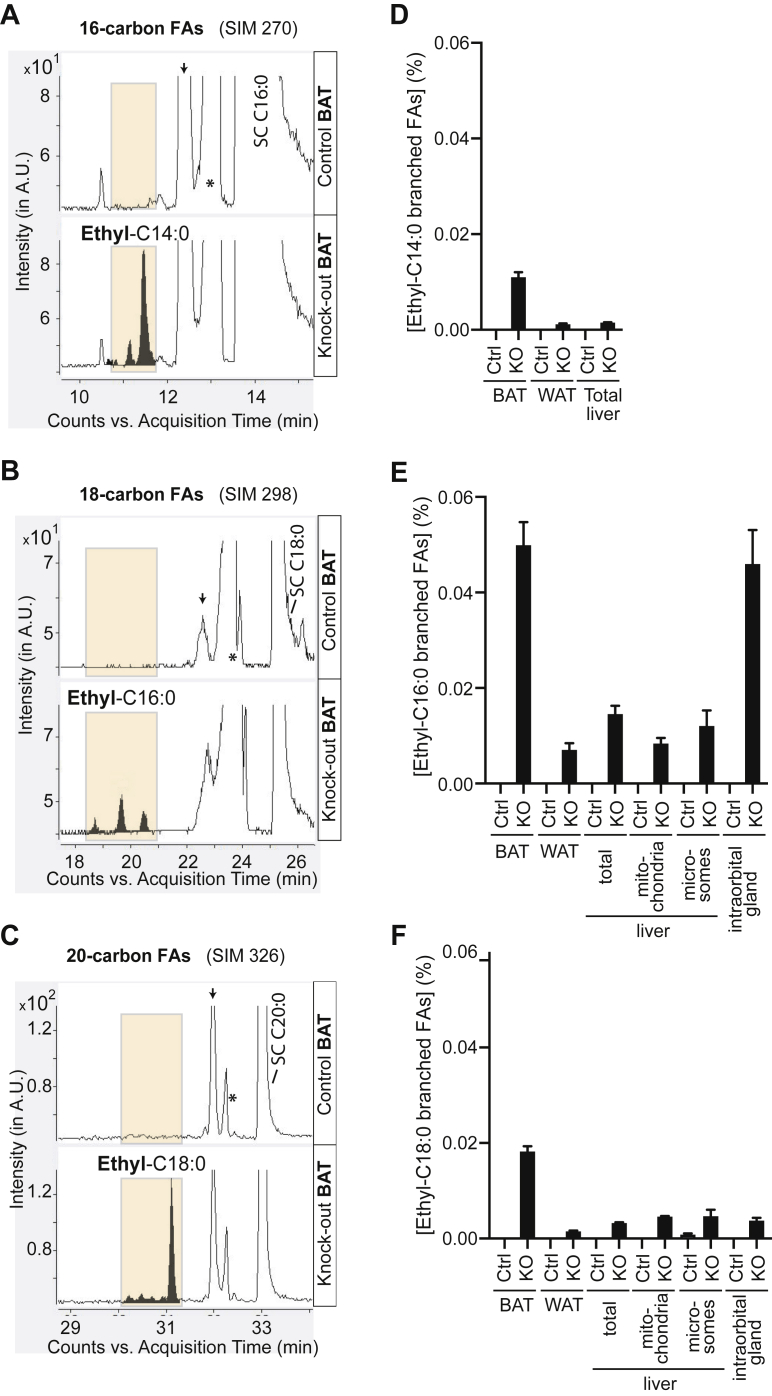
**Ethyl-branched fatty acids are exclusively found in ECHDC1 KO mouse tissues**. *A–C*, GC-MS elution profiles of (*A*) 16-carbon FAs (SIM 270), (*B*) 18-carbon FAs (SIM 298), and (*C*) 20-carbon FAs (SIM 326) after derivatization to methyl esters were acquired from samples of brown adipose tissue. Ethyl-branched FAs are filled in *black* and highlighted in *ocher*. *D–F*, quantitative analysis of ethyl-C14:0 (*D*), ethyl-C16:0 (*E*), and ethyl-C18:0 (*F*) branched-chain FAs in different tissues and liver mitochondria. Metabolites were quantified after derivatization to methyl esters. Results are expressed as the percentage of the sum of C14:0, C15:0, C16:0, C17:0, and C18:0 and are shown as the means ± SEM of at least four mice per group (except for intraorbital glands, where n = 3). *Small arrows* indicate the position of *iso*-methyl-branched fatty acids. *Asterisks* indicate the presence of a contaminant peak corresponding to the M + 2 of the corresponding monounsaturated straight-chain fatty acid. A.U., arbitrary units; BAT, brown adipose tissue; ECHDC1, ethylmalonyl-CoA decarboxylase; FAs, fatty acids; SC, straight chain; SIM, single ion monitoring; WAT, white adipose tissue.

To detect branched-chain FAs in different tissues, we took advantage of two complementary approaches. First, we analyzed fatty acid methyl esters (FAMEs) by GC-MS. Samples were obtained by methanolysis and transesterification of extracted lipid metabolites. This allowed us to analyze free FAs and FAs released from triglycerides or phospholipids, but not from cholesterol esters, sphingolipids, or proteins. We had previously found that ethyl-branched FAs elute before the corresponding straight-chain (SC) FAs (2). We observed several peaks corresponding to FAs with 16-, 18-, and 20-carbons in ECHDC1 knockout tissues, which were completely absent in heterozygous controls (Fig. 2*A*–*C*). For all three chain lengths, the first of these knockout specific peaks most likely represents the 2-ethyl-branched FA, since it strictly coelutes with an m/z 102 ion that is known to be formed by a McLafferty rearrangement of 2-ethylbranched FAs (11) (Fig. S1, *A* and *B*). This was most apparent in samples from BAT (Fig. S1*A*) and intraorbital glands comprising both Harderian and intraorbital lacrimal glands (Fig. S1*B*), but also detectable at lower levels in WAT and liver of ECHDC1-deficient mice (Fig. S1*B*).

The localization of the ethyl-group in the other peaks could not be attributed by analysis of FAMEs with GC-MS. To overcome this limitation, we also derivatized FAs with 3-pyridyl-carbinol (“picolinyl”), which allows one to identify the location of the ethyl-branch (Fig. S2). Using this second approach, we found that ethyl-branches were mainly positioned on the ω-4 (10-ethyl-C14:0, 12-ethyl-C16:0, and 14-ethyl-C18:0, Fig. S2, *A–C*) and the ω-6 (8-ethyl-C14 and 10 ethyl-C16:0, Fig. S2, *A* and *B*) carbons.

The combined signal of all ethyl-C16:0 FAs represented approximately 0.05 % of the signal of total straight chain FAs (*i.e.*, the sum of C14:0, C15:0, C16:0, C17:0, and C18:0) in BAT (Fig. 2*E*) and in the intraorbital glands (Fig. 2*E*). This signal was about 5-fold higher than in WAT or liver samples (Fig. 2*E*). Levels of ethyl-C14:0 and ethyl-C18:0 FAs (Fig. 2, *D* and *F*) were also the highest in BAT. Of note, the relative abundance of ethyl-branched FAs in the liver was comparable in total cellular lysates, mitochondrial extracts, and extracts from the endoplasmic reticulum (microsomes), suggesting that ethyl-branched FAs are incorporated into lipids to the same extent in these compartments (Fig. 2, *E* and *F*).

### Fatty acids with methyl-branches on even-numbered carbons are detectable in normal mouse tissues and increased in ECHDC1 knockout intraorbital gland tissue

**Figure 3:**
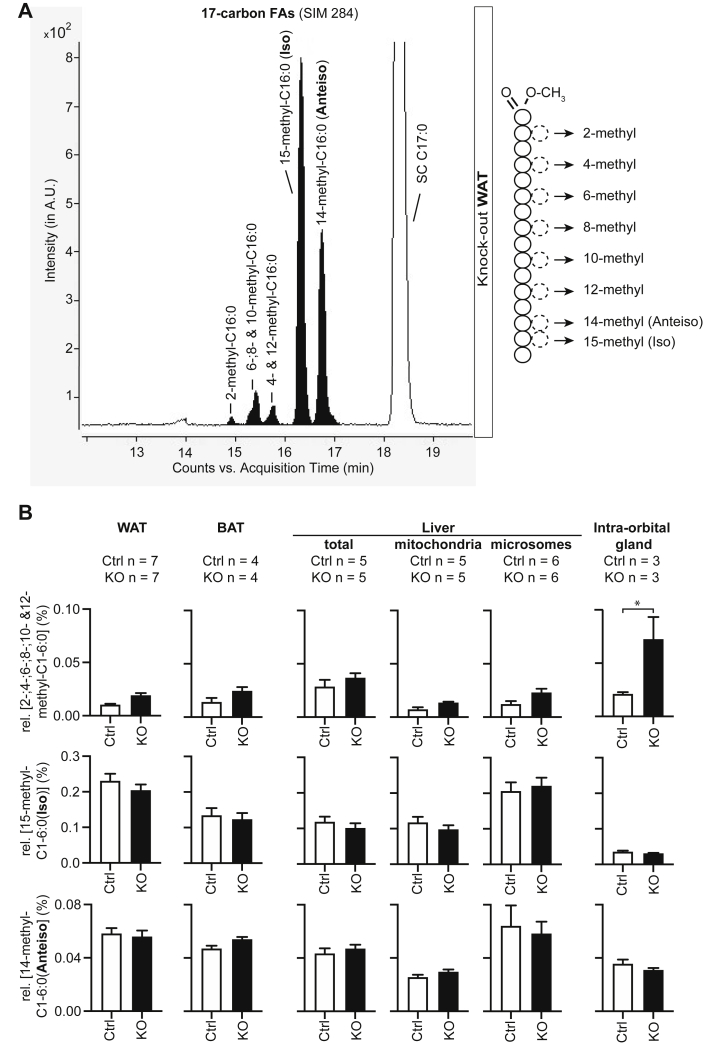
**Methyl-branched fatty acids are little or not increased in ECHDC1 KO tissues**. *A*, GC-MS elution profile of 17-carbon FAs (SIM 284) in white adipose tissue showing the position of the detected methyl-branched FAs (filled in *black*) and of the isomeric straight chain FA. *B*, quantitative analysis of the sum of 2-, 4-, 6-, 8-, 10-, and 12-methyl-branched (*upper panels*), *iso*-branched (*middle panels*), and *anteiso*-branched (*lower* panels) FAs in white adipose tissue, brown adipose tissue, liver, and intraorbital glands (as percentage of the sum of C14:0, C15:0, C16:0, C17:0, and C18:0). ECHDC1, enzyme ethylmalonyl-CoA decarboxylase; FAs, fatty acids.

A recent study highlighted that branched-chain FAs are formed under physiological conditions, when FA synthesis starts with CoA derivatives containing a side chain such as 2-methyl-propionyl-CoA (= isobutyryl-CoA), 3-methyl-butyryl-CoA (= isovaleryl-CoA), and 2-methyl-butyryl-CoA. This leads to methyl branches on the penultimate (*iso* forms) and ante-penultimate carbon (*anteiso* form) (1). In contrast, the presence of branched-chain FAs with methyl-branches on other carbons has not been evaluated. We have previously optimized the quantification and identification of these methyl-branched FAs based on work from Nicolaides and colleagues (2, 12). As previously observed by Wallace and colleagues (1), we also detected *anteiso* and *iso* forms of methyl branched FAs that were of comparable abundance in wild-type and knockout samples (Fig. 3, *A* and *B*). However, in addition we clearly observed methyl-branched FAs carrying methyl-branches on even-numbered carbon atoms (eluting before the *anteiso* and *iso* forms in Fig. 3*A*), which can be synthesized when methylmalonyl-CoA is used instead of malonyl-CoA during FA synthesis. These FAs were increased 2.5-fold in the intraorbital glands of ECHDC1 knockout mice, whereas little or no increase was observed in other organs (Figs. 3*B* and S3, *A* and *B*). Of note, we had previously reported that inactivation of ECHDC1 in two different adipocyte cell lines (3T3-L1 and L929) leads to a two- to fivefold increase of methylmalonyl-CoA-derived methyl-branched FAs ((2) and Fig. S3*C*). This indicates that ECHDC1 contributes little to maintain low cytosolic methylmalonyl-CoA levels in most mouse tissues. Lower levels of cytosolic propionyl-CoA in tissues are the most likely cause for this difference between adipocyte cell lines and mouse tissues. Cytosolic propionyl-CoA is not only the precursor of methylmalonyl-CoA but also required for the synthesis of fatty acids with uneven carbon numbers. Therefore, the proportion of FAs with an uneven carbon number can be used to estimate cytosolic propionyl-CoA concentrations. We observed that these fatty acids were much lower in mouse tissues than in cell lines. For example, relative to the sum of all total straight chain saturated FAs, the most common form with uneven carbon numbers, C17:0, only represented ≈0.6 % in WAT as compared with 2.5 % in 3T3-L1 cells and 10.4 % in L929 adipocyte cells (data not shown).

### Ethyl-branched lipids are incorporated into complex lipids and accumulate in ECHDC1 deficient tissues

**Figure 4:**
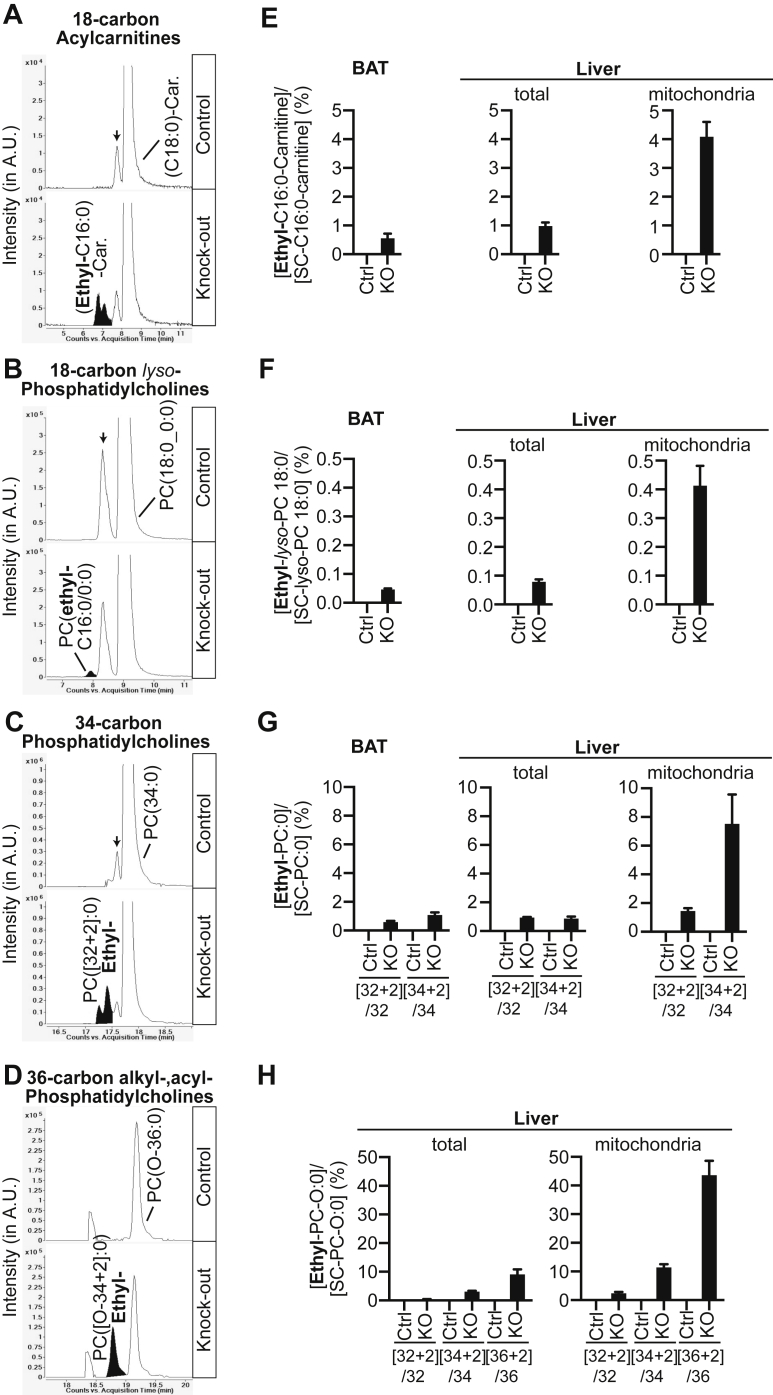
**Presence of ethyl-branched fatty acids in acylcarnitines, (*lyso*)phosphatidylcholine, and plasmanylcholine in ECHDC1 KO tissue extracts**. *A–D*, elution profile of (*A*) 18-carbon acylcarnitine species (C25H49NO4, *[M + H]+ 428.3734*), (*B*) 18-carbon *lyso*-phosphatidylcholine species (C26H54NO7P, *[M + H]+ 524.3711*), (*C*) 34-carbon phosphatidylcholine species (C42H84NO8P, *[M + H]+ 762.6006*), and (*D*) 36-carbon plasmanylcholine (acyl, alkyl phosphatidylcholines) species (C44H90NO7P, [M + H]+ 776.6528)). Putative ethyl-branched containing species are filled in *black*. *Small arrows* indicate unknown isomers. *E–H*, quantitation of ethyl-branched species in (*E*) 18-carbon acylcarnitine, (*F*) 18-carbon *lyso*-phosphatidylcholine, (*G*) 34-carbon phosphatidylcholine, and (*H*) 36-carbon plasmanylcholine in brown adipose tissue (BAT), total liver, and liver mitochondria. Values are the means ± SEM for four (BAT) or five (liver and liver mitochondria) samples of control and ECHDC1 KO mice. Values are expressed as the percentage of the non-ethyl-branched counterpart species containing two carbons less in total. A.U., arbitrary units; ECHDC1, ethylmalonyl-CoA decarboxylase.

Having established that ECHDC1 knockout mice accumulate ethyl-branched FAs, we wanted to determine if they are incorporated into complex lipids. To this end, we analyzed hydrophobic metabolites from mouse organs using an LC-MS method that allows one to separate a large variety of lipids. Given the combinatorial complexity of lipids that contain one or more FAs with different chain lengths and desaturations, we relied on a nontargeted analysis to identify peaks that are present in knockout and absent in control samples. Several peaks were identified that were much more abundant in knockout than in wild type mouse samples (Fig. 4, *B*–*D*). Notably, these peaks eluted just before a peak that was unchanged between knockout and control samples, suggesting that these peaks are isomers (Figs. 4 and S4). The systematic extraction of m/z values corresponding to these compounds +C_2_H_4_ or −C_2_H_4_ (*i.e.*, the chemical formula of an “ethyl branch”) combined with a systematic m/z extraction of other lipid classes (including phosphatidylethanolamines; acylcarnitines (Fig. 4*A*); ceramides; sphingomyelins and triglycerides (Fig. S4)) uncovered several additional compounds that were not initially picked up in the untargeted approach.

Based on their m/z, these compounds were identified as long-chain acylcarnitines, glycerophospholipids, and triglycerides. We hypothesized that the additional peaks of octadecanoylcarnitine corresponded to isomers of ethyl-branched-hexadecanoylcarnitine that were not resolved in LC (Fig. 4*A*). This is compatible with the observation that ethyl-branched hexadecanoate was the most abundant ethyl-branched FA detected in tissues (Fig. 2*E*).

Among the knockout-specific glycerophospholipid peaks, we observed *lyso*-phosphatidylcholine PC 18:0 isomers (Fig. 4*B*), phosphatidylcholine PC 34:0 and PC 36:0, isomers (Fig. 4*C* and data not shown), and alkyl-acyl phosphatidylcholine (PC O-34:0, PC O-36:0, and PC O-38:0) isomers (Fig. 4*D* and data not shown). Interestingly, no additional forms were detected that could correspond to ethyl-branched phosphatidylethanolamines PE 34:0 & PE 36:0.

Alkyl-acyl phosphatidylcholines are also called plasmanylcholines and represent a subcategory of ether-phospholipids (Fig. 4*D*). The knockout-specific plasmanylcholine peaks presumably corresponded to a form containing a putative ethylbranched acyl-chain (or fatty alcohol). Of note, we did not observe any knockout specific accumulation of the corresponding plasmenylcholines (PC P-36:1 and PC P-38:1) (not shown), their monounsaturated counterparts that are also called plasmalogens. This observation is consistent with the fact that plasmenylcholines do not arise from the desaturation of plasmanylcholine, but from the metabolism of plasmenylethanolamine (formed by desaturation of plasmanylethanolamine). Thus, the absence of knockout-specific peaks of plasmanyl- or plasmenylethanolamines (not shown) likely explains the absence of knockout-specific plasmenylcholine isomers. Yet, this line of reasoning does not explain the absence of knockout-specific phosphatidylethanolamine or plasmanylethanolamine peaks. Thus, it remains conceivable that ethyl-branched FAs are either not incorporated in or removed from ethanolamine-containing glycerophospholipids.

Knockout-specific triglyceride species were also observed in BAT (Fig. S4: TG 52:1, TG 52:0, and TG 54:0). All these additional species were putative ethyl-branched (rather than methyl-branched) FA containing lipids, since they were completely absent in control tissues, and they all contained an even number of carbons in their acyl-groups.

To compare the abundance of complex lipids with ethyl-branches between organs and subcellular compartments, we divided the area under the curve of these metabolites by the area under the curve of the nonbranched counterpart containing two carbons less. This led to two interesting observations. First, we observed that the abundance of knockout-specific acylcarnitines, *lyso*-phosphatidylcholines, and phosphatidylcholine was comparable between the liver and BAT (Fig. 4, *E–G*). Thus, the higher amount of total ethyl-branched FAs in BAT (Fig. 2*E*) is likely due to larger amounts of triglycerides in this tissue. Curiously, we also observed that mitochondria were enriched in ethyl-branched FA containing complex lipids (Fig. 4, *E–H*). This was most striking for PC [O-36 + 2]:0, which represented 46% of the corresponding nonbranched compound (with two less carbons) (Fig. 4*H*).

### Medium ethyl-branched acyl chains and derivatives accumulate in urine and tissues of ECHDC1-deficient mice

**Figure 5:**
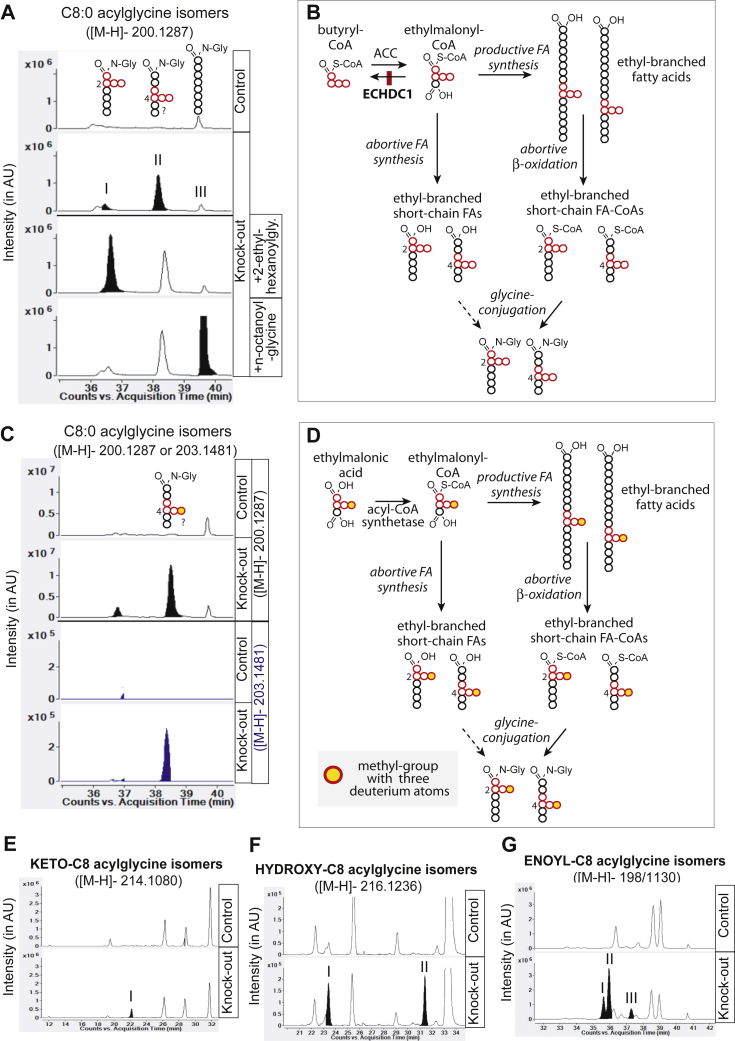
**Ethyl-branched hexanoylglycine and derivatives accumulate in urine from ECHDC1-deficient mice**. *A*, extracted ion chromatogram (EIC) of 8-carbon acylglycine isomers in urine and spike-in experiments allowing partial identification: peak I: 2-ethyl-hexanoylglycine; peak II: 4-ethylhexanoylglycine (presumed); peak III: n-octanoylglycine. *B*, presumed metabolic origin of ethyl-branched acylglycine compounds containing an 8-carbon fatty acid. *C*, EIC of 8-carbon acylglycine isomers in urine and corresponding M + 3 isotopologues, after feeding mice with labeled ethylmalonate. *D*, presumed metabolic origin of ethyl-branched acylglycine compounds after feeding the mice with labeled ethylmalonate. *E–G*, EIC of 8-carbon (*E*) keto-acylglycine isomers, (*F*) hydroxy-acylglycine isomers, and (*G*) enoyl-acylglycine isomers. Presumed ethyl-branched species are filled in *black*. Numbering of peaks on the chromatograms corresponds to the numbering in Table 1. ECHDC1, ethylmalonyl-CoA decarboxylase.

The presence of ethyl-branches in the FAs could lead to problems during synthesis and degradation. Thus, products of abortive FA synthesis and degradation might accumulate or be excreted in the urine. When we analyzed hydrophilic metabolites with an untargeted, negative mode, LC-MS approach, which is particularly sensitive for carboxylic acids, we found more than 100 compounds that were strongly increased in concentration (most often by more than 20-fold) in the urine of ECHDC1 knockout mice. Among the top 120 uncovered metabolites, more than 20 corresponded in mass to glycine and taurine conjugates of FA derivatives with a total of 8, 10, and 12 carbons. Others were found after systematic analysis of m/z corresponding to all the glycine or taurine conjugates containing acyls with 4–20 carbons at various states of oxidation (Table 1). We found saturated, monounsaturated (enoyl-), hydroxylated, and keto-group-containing putative branched-chain FAs conjugated to glycine, which all could be derived from abortive FA degradation or synthesis (Table 1; Figs. 5 and S5, *E–G*). In addition, several taurine-conjugated FAs, unconjugated FAs, and dicarboxylic acid isomers were increased (Table 1 and Fig. S6).

**Table 1:** Medium acyl chains accumulation in ECHDC1-deficient mouse urine

Carboxylic acids		Conjugation	Carbons	*[M-H]*−	Formula	Numbering (RT)	CTRL (n = 7)		ECHDC1 KO (n = 8)		KO/CTRL fold change
							Mean	SD	Mean	SD	
Monocarboxylic	Keto	Glycine	8	214.1080	C10H17NO4	I (22.2′)	6.62E-06	±2.30E-06	1.68E-04	±1.00E-04	25.38
			9	228.1236	C11H19NO4	I (23.4′)	ND		4.76E-06	±3.71E-06	Only in KO
						II (28.5′)	7.46E-07	±6.45E-07	7.21E-06	±2.27E-06	9.67
			10	242.1392	C12H21NO4	I (32.8′)	ND		3.61E-05	±3.79E-05	Only in KO
						II (33.5′)	8.28E-06	±6.12E-06	5.48E-05	±2.08E-05	6.62
						III (33.9′)	4.49E-05	±2.11E-05	2.48E-04	±3.91E-05	5.54
						IV (34.0′)	1.14E-04	±3.52E-05	9.42E-04	±4.19E-04	8.27
						V (34.5′)	5.20E-07	±1.38E-06	4.15E-04	±2.09E-04	798.90
						VI (39.5′)	2.19E-05	±8.82E-06	3.04E-04	±1.00E-04	13.92
			12	256.1548	C13H23NO4	I (38.6′)	ND		5.19E-05	±9.05E-05	Only in KO
						II (39.3′)	ND		2.90E-05	±2.43E-05	Only in KO
						III (40.8′)	2.36E-06	±2.39E-06	1.80E-05	±2.08E-05	7.60
		Taurine	8	240.1283	C10H19NO5S	I (26.5′)	7.54E-06	±2.29E-06	3.03E-05	±1.05E-05	4.03
			10	268.1596	C12H23NO5S	I (39.5′)	6.34E-06	±4.92E-06	5.38E-05	±1.77E-05	8.49
	Hydroxy	Glycine	8	216.1236	C10H19NO4	I (23.5′)	8.95E-06	±4.78E-06	7.04E-05	±2.81E-05	7.87
						II (31.5′)	ND		7.28E-05	±3.67E-05	Only in KO
			10	244.1549	C12H23NO4	I (33.2′)	1.26E-05	±5.10E-06	6.53E-05	±4.47E-05	5.18
						II (33.6′)	6.22E-06	±1.92E-06	6.72E-05	±2.22E-05	10.80
						III (34.9′)	4.77E-06	±2.91E-06	2.81E-04	±8.54E-05	59.02
						IV (35.1′)	4.06E-05	±1.53E-05	1.76E-04	±6.72E-05	4.34
						V (35.7′)	1.93E-05	±7.70E-06	1.65E-04	±6.81E-05	8.55
						VI (40.4′)	1.42E-06	±5.04E-07	2.54E-04	±1.03E-04	178.42
			12	272.1862	C14H27NO4	I (41.0′)	ND		5.57E-06	±6.36E-06	Only in KO
						II (41.1′)	ND		8.57E-06	±1.66E-05	Only in KO
						III (41.8′)	ND		5.94E-06	±1.01E-05	Only in KO
		Taurine	10	294.1375	C12H25NO5S	I (35′)	6.88E-07	±3.17E-07	7.70E-06	±2.10E-06	11.19
	Unsaturated	Glycine	8	198.1130	C10H17NO3	I (35.7′)	4.02E-06	±7.82E-06	4.77E-04	±1.64E-04	118.48
						II (36′)	2.01E-05	±1.33E-05	1.08E-03	±4.22E-04	53.57
						III (37.4′)	1.32E-05	±8.84E-06	2.23E-04	±1.04E-04	16.86
			9	212.1287	C11H19NO3	I (28.5′)	ND		2.40E-06	±9.09E-07	Only in KO
			10	226.1443	C12H21NO3	I (41.2)	2.54E-05	±8.73E-06	2.47E-04	±2.44E-04	9.72
						II (41.5′)	2.27E-06	±1.37E-06	7.42E-05	±9.46E-05	32.70
		Taurine	8	248.0957	C10H19NO4S	I (35.6′)	ND		1.00E-05	±3.75E-06	Only in KO
						II (35.9′)	5.93E-07	±4.78E-07	2.98E-05	±1.28E-05	50.21
			9	262.1113	C11H21NO4S	I (25.3′)	5.62E-07	±3.30E-07	1.51E-05	±4.05E-06	26.86
			10	276.1270	C12H23NO4S	I (41.1′)	7.53E-06	±2.06E-06	2.90E-04	±1.28E-04	38.52
			12	290.1426	C13H25NO4S	I (42.5′)	5.30E-06	±1.99E-06	1.83E-05	±3.27E-05	3.46
	Saturated	Glycine	8	200.1287	C10H19NO3	I (37.2′) 2-ethylhexanoyl-	1.25E-04	±4.58E-05	6.50E-04	±3.42E-04	5.20
						II (38.9′)	8.79E-05	±3.24E-05	3.08E-03	±8.44E-04	35.04
			9	214.1443	C11H21NO3	I (29′)	ND		4.21E-06	±1.46E-06	Only in KO
			10	228.1600	C12H23NO3	I (41.8′) 4-ethylhexanoyl-	3.84E-06	±2.97E-06	2.14E-04	±2.63E-04	55.81
		Taurine	8	250.1113	C10H21NO4S	I (37.0′)	3.63E-07	±6.86E-07	1.46E-05	±5.38E-06	40.21
						II (38.9′)	2.31E-07	±1.67E-07	6.57E-05	±4.62E-05	283.96
			10	278.1426	C12H25NO4S	I (41.7′)	1.55E-06	±2.02E-06	2.06E-04	±3.73E-04	132.49
		Glucuronide	8	319.1393	C14H24O8	I (38.9′)	2.99E-06	±2.57E-06	4.67E-05	±5.02E-05	15.61
	Keto-unsaturated	Glycine	10	240.1236	C12H19NO4	C10:1-keto I (32.1′)	1.91E-07	±5.04E-07	6.26E-05	±6.01E-05	328.31
						C10:1-keto II (32.8′)	ND		1.33E-05	±9.17E-06	Only in KO
			12	268.1549	C14H23NO4	C12:1-keto I (36.9′)	5.01E-06	±2.26E-06	9.44E-05	±6.69E-05	18.84
						C12:3-keto I (39.5′)	2.13E-07	±2.87E-07	6.83E-06	±3.24E-06	31.98
Dicarboxylic	Saturated	Glycine	8	230.1028	C10H17NO5	I (21.4′)	ND		4.03E-05	±1.95E-05	Only in KO
			10	258.1341	C12H21NO5	I (29.2′)	1.24E-06	±3.16E-07	1.82E-04	±7.41E-05	146.71
	Unsaturated			256.1185	C12H19NO5	I (27′)	2.46E-06	±1.40E-06	1.34E-04	±5.72E-05	54.52
						II (28.8′)	3.41E-06	±1.88E-06	3.14E-04	±1.55E-04	91.93
	Saturated	Taurine	8	280.0855	C10H19NO6S	I (22.4′)	1.68E-06	±8.65E-07	1.53E-04	±1.82E-04	91.54
			10	308.1168	C12H23NO6S	I (29.6′)	2.20E-06	±1.71E-06	1.64E-04	±1.62E-04	74.67
	Unsaturated			306.1011	C12H21NO6S	I (29.1′)	1.88E-06	±1.47E-06	1.71E-04	±6.10E-05	91.36
						II (30.3′)	6.92E-07	±8.32E-07	1.68E-05	±6.49E-06	24.34
Monocarboxylic	Saturated	Free	9	157.1229	C9H18O2	C9 (29.5')	ND		5.83E-06	±1.21E-06	Only in KO
	Unsaturated			155.1072	C9H16O2	C9:1 I (28.4′)	ND		5.07E-05	±9.83E-06	Only in KO
						C9:1 II (28.8′)	ND		2.17E-06	±8.47E-07	Only in KO
						C9:1 III (29.2′)	ND		3.41E-06	±6.96E-07	Only in KO
			10	167.1072	C10H16O2	C10:2 I (34.6′)	3.07E-08	±8.13E-08	3.38E-06	±1.40E-06	109.93
						C10:2 II (39.5′)	ND		2.74E-05	±9.35E-06	Only in KO
				165.0916	C10H14O2	C10:3 I (32.1′)	ND		1.36E-05	±1.46E-05	Only in KO
						C10:3 II (32.8′)	ND		2.43E-06	±9.67E-07	Only in KO
Amide	Saturated		9	156.1388	C9H19NO	Nonanamide (38.9′)	1.14E-06	±3.20E-07	4.97E-05	±1.42E-05	43.58
Dicarboxylic	Saturated	Free	6	145.0501	C6H10O4	I (15.5′)	1.50E-05	±1.05E-05	5.25E-05	±1.66E-05	3.50
			8	173.0814	C8H14O4	I (20.1′)	ND		1.91E-05	±1.64E-05	Only in KO
						II (21.1′)	ND		4.44E-05	±2.91E-05	Only in KO
			10s	201.1127	C10H18O4	I (29.5′)	ND		6.86E-04	±1.44E-04	Only in KO
			12	229.1440	C12H22O4	I (31.6′)	ND		6.60E-06	±6.08E-06	Only in KO
		Sugar?		404.1927	C18H31NO9 (?)	Unknown (30.6′)	ND		2.50E-04	±1.58E-04	Only in KO

Abbreviations: ND, not detectable; RT, retention time.

**Figure 6:**
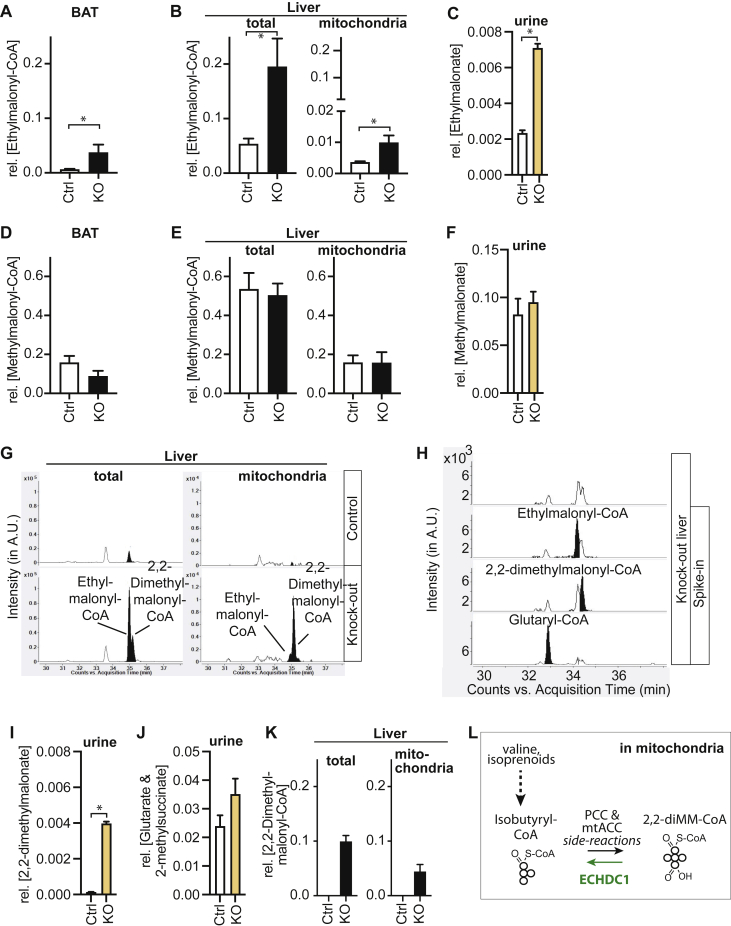
**Ethylmalonyl-CoA and 2,2-dimethylmalonyl-CoA accumulate in ECHDC1-deficient tissues**. *A–F*, LC-MS quantitative analysis of (*A*) ethylmalonyl-CoA in brown adipose tissue (BAT), (*B*) ethylmalonyl-CoA in the liver, (*C*) ethylmalonate in urine, (*D*) methylmalonyl-CoA in the liver, (*E*) methylmalonyl-CoA in the liver, and (*F*) methylmalonate in urine. *G*, elution profiles of ethylmalonyl-CoA in the liver, *[M-H]*^*−*^*880.1396.H,* spike-in experiments allowing the identification of isomeric peaks, *[M-H]*^*-*^*880.1396. I–K*, LC-MS quantitative analysis of (*I*) 2,2-dimethylmalonate in urine, (*J*) glutarate and methylsuccinate in urine (not resolved using our LC-MS method), and (*K*) 2,2-dimethylmalonyl-CoA in the liver. *L*, presumed origin of 2,2-dimethylmalonyl-CoA in mitochondria. Values are the means ± SEM for four (BAT) or five (liver and liver mitochondria) or at least seven (urine) samples of control and ECHDC1 KO mice. Values were normalized to the sum of all extracted peaks for each sample after alignment and automatic integration using MassHunter Mass Profiler software (Agilent). ECHDC1, ethylmalonyl-CoA decarboxylase.

The type of the carboxylic acid is indicated in the first column and their carbon number in column 4. Isomers are numbered (I, II, …) according to their order of elution. Values are means of the indicated number of samples, normalized to the sum of all extracted peaks for each sample after alignment and automatic integration using MassHunter Mass Profiler software (Agilent). Dicarboxylic acids with 5 carbons are shown in Figure 6.

Remarkably, increases in conjugates with FAs containing an uneven carbon number (7, 9, or 11) were either not observed (7 or 11 carbons) or in the case of 9 carbons much less abundant than those observed with 8 or 10 carbon FAs. These observations suggested that the increased FAs contained an ethyl-branch rather than methyl-branches.

To corroborate that this was indeed the case, we used synthetic standards and followed the incorporation of isotopically labeled ethylmalonate into these compounds. This is illustrated in Figures 5*D* and S5*D* for glycine conjugates of FAs with eight and ten carbons. The extracted ion chromatograms corresponding to the m/z of a glycine conjugate with an eight-carbon FA (m/z 200.1287, Fig. 5*A*) revealed three isomers. Two isomers (labeled with I and II) were highly increased in urine of ECHDC1 KO mice while the third peak (III) was unchanged. Using synthetic standards, we found that peaks I and III coeluted with 2-ethylhexanoylglycine and n-octanoylglycine, respectively (Fig. 5*A*). Using spiked-in standards and assuming that electrospray ionization of ethylhexanoylglycine and n-octanoylglycine is comparable, we estimated the urinary excretion to be about 0.014 mol/mol creatinine, *i.e.*, ≈50 nmol ethylhexanoylglycine per day.

For the C10:0-acylglycines, we found one major increased peak (at ≈ 41–42 min) in the urine of ECHDC1 KO mice (Fig. S5*A*). This peak coeluted with a synthetic standard of 4-ethyloctanoylglycine and could be differentiated from 2-ethyloctanoylglycine and n-decanoylglycine, which eluted distinctly earlier and later, respectively (Fig. S5*A*).

To ensure that the putative ethyl-branched acylglycines found in urine of ECHDC1 KO mice were derived from ethylmalonyl-CoA, we fed heterozygous control and ECHDC1-deficient mice with ethylmalonate carrying three deuterium atoms on the ethyl-side chain ([ethyl-d3]-ethylmalonate). We analyzed by LC/MS urine samples collected after 4 h. This revealed an increase of the M+3 isotopologues of the putative ethyl-branched compounds (Figs. 5*C* and S5*C*) whose abundance was increased in ECHDC1 knockout mice samples.

For the C8:0-acylglycine isomers (Fig. 5*C*), a strong incorporation was observed in peak II, a weaker one in peak I, but none in peak III. This supports the idea that peaks I and II are both ethylbranched acylglycines, whereas peak III corresponds to n-octanoylglycine. Because peak I coeluted with 2-ethylhexanoylglycine (Fig. 5*A*), peak II most likely corresponds to 4-ethylhexanoylglycine, the only other possible isomer of octanoylglycine resulting from ethylmalonyl-CoA incorporation during chain elongation (Fig. 5*B*). Of note, the abundance of the labeled peaks (M+3) remained very low (*i.e.*, 1/60,000 of the corresponding unlabeled peak) indicating that only a fraction of the cellular ethylmalonyl-CoA pool was labeled. The major peak found among C10:0-acylglycines in ECHDC1 KO mice samples was also labeled (although weakly) by deuterated ethylmalonate (Fig. S5*C*), which supports our structural assignment as 4-ethyloctanoyl-glycine in the spike-in experiment (Fig. S5*A*).

Taken together, based on the coelution with synthetic standards and deuterium incorporation from [ethyl-d3]-ethylmalonate, we conclude that ECHDC1 knockout mice excrete glycine-adducts of ethyl-branched FAs. Several of these glycine adducts were also detectable in tissue samples, particularly in the liver in accordance with the fact that glycine N-acyltransferase (GLYAT) expression is the highest in this organ. Derivatives of ethyl-branched FAs could be the result of failed attempts to synthesize ethyl-branched FAs or to degrade these compounds.

### ECHDC1 knockout mice accumulate not only cytosolic ethylmalonyl-CoA, but also mitochondrial 2,2-dimethylmalonyl-CoA

As expected, LC-MS analysis of CoA esters in BAT and liver revealed that, compared with controls, ECHDC1 KO mouse tissues had significantly higher ethylmalonyl-CoA levels (Fig. 6, *A* and *B*). Consistent with this, we observed that knockout mice excreted significantly more ethylmalonic acid in urine (Fig. 6*C* in LC-MS, Fig. S7 in GC-MS). In contrast, there was no significant difference in methylmalonyl-CoA levels between control and ECHDC1 knockout tissues (Fig. 6, *D* and *E*) and no difference in urinary methylmalonic acid excretion (Fig. 6*F* in LC-MS, Fig. S7 in GC-MS).

Curiously, the LC-MS chromatogram of the predicted m/z of ethylmalonyl-CoA ([M-H]^−^ 880.1396; C_27_H_44_N_7_O_19_P_3_S) showed an additional peak eluting immediately after ethylmalonyl-CoA exclusively in knockout tissues (Fig. 6*G*). Using a synthetic standard, we tentatively identified this peak as 2,2-dimethylmalonyl-CoA (Fig. 6*H*). Consistent with this identification, we detected elevated levels not only of ethylmalonate but also of 2,2-dimethylmalonate in the urine of ECHDC1 deficient mice, (Fig. 6*I* by LC-MS, Fig. S7 by GC-MS).

Interestingly, while we observed a much higher ratio of ethylmalonyl-CoA to 2,2-dimethylmalonyl-CoA in total liver extract, we observed more 2,2-dimethylmalonyl-CoA than ethylmalonyl-CoA in liver mitochondrial extracts (Figs. 6*G*, and 6*K* *versus* 6*B*). 2,2-Dimethylmalonyl-CoA can be produced by carboxylation of isobutyryl-CoA, a mitochondrial intermediate in the degradation of valine, iso-branched FAs, or isoprenoids (10, 13). Published proteomic analyses of purified mitochondria had previously revealed some ECHDC1 protein in mitochondria (14), indicating that this protein may prevent accumulation of mitochondrial 2,2-dimethylmalonyl-CoA. This raises the question why loss of ECHDC1 does not lead to a stronger increase in mitochondrial ethylmalonyl-CoA levels (Fig. 6*B*, right panel). Previous work indicates that mitochondrial ethylmalonyl-CoA may be partially converted to methylsuccinyl-CoA in a side reaction of methylmalonyl-CoA mutase (15). Consistent with this reasoning, we observed a moderate increase of 2-methylsuccinate by GC-MS analysis (Fig. S7), which could not be resolved from glutarate by LC-MS (Fig. 6*J*).

Overall, these data underline that the modest specificity of ECHDC1 allows cells to limit the accumulation of a variety of branched-chain dicarboxyl-CoA compounds, thereby preventing a disturbance in FA synthesis.

## Discussion

### Presence of unique long-chain ethyl-branched FAs and high relative enrichment in mitochondrial plasmanylcholine species

The most striking new observation is that ECHDC1 inactivation leads to the appearance or very strong increases (>20-fold) of FAs that are confirmed or extremely likely to be ethyl-branched species.

First, we found evidence for the presence of several peaks of ethyl-branched-C14:0, -C16:0, and -C18:0 FAs (isomers of 16-, 18-, and 20-carbon FAs, respectively), mostly in BAT. The elution profile of these peaks did not correspond to monomethyl-branched-C17:0 or dimethyl-branched-C16:0 according to our previous results in cell models (Fig. S1*C*) and we could show, for one of the peaks, coelution with an m/z 102 ion (Fig. S1, *A* and *B*), which is the characteristic McLafferty rearrangement of FAs with an ethyl branch on C_2_ (Fig. S1*A*, right panel). Furthermore, “picolinyl” derivatization revealed that the position of the branches was on ω-4 and ω-6 carbons for the predominant peaks (Fig. S2). This indicates that the ethylmalonyl-CoA incorporation occurred during the second or the third step of FA synthesis, suggesting that most ethyl branches are incorporated by FA synthase and not by elongases.

### Based on the signal intensity in GC-MS, ethyl-branched FA represented a small fraction of all fatty acids, likely less than 0.05 % in brown adipose tissue

We also found evidence that abnormal, most likely ethyl-branched FAs are incorporated into complex lipids. This was most apparent for phosphatidylcholine species in several tissues and for plasmanylcholine species in the liver, especially in mitochondria. Two arguments strongly suggest that the observed species correspond to ethyl-branched FAs rather than methyl-branched FAs. First, no such compounds were observed with an odd number of carbons for the two acyl chains contained in phosphatidylcholine and plasmanylcholine, which would have been expected if one of the acyl chain was mono-methyl-branched. Second, these lipids were strongly increased in samples from knockout mice, whereas methyl-branched FAs were virtually unchanged in most analyzed tissues.

Strikingly, the relative abundance of ethyl-branched isomers compared with their linear counterparts (*i.e.*, with two carbon less due to absence of the ethyl branch) was much higher, reaching up to 46 % for the plasmanyl forms (Fig. 4*H*). This high relative enrichment is in stark contrast to the relatively small proportion of ethyl-branched FAs among all FAs even in knockout cells. The most likely explanation seems to be a lack of metabolism of ethyl-branched lipids by phospholipase A2 due to crowding caused by the ethyl-branch. Though this has not been tested with ethyl-branched FAs, it has indeed been shown that methyl-branched FAs are only slowly metabolized by phospholipase A2, and that they may even behave as inhibitors (16). As such, once incorporated into complex lipids, some ethyl-branched FAs might be very difficult to remove.

Interestingly, some ethyl-branched plasmanylcholine species were particularly high in liver mitochondria. Given the relatively subtle increase in mitochondrial ethylmalonyl-CoA, it seems unlikely that these lipids are the result of mitochondrial FA synthesis. An alternative hypothesis would be that this particular class of lipids is formed in this organelle or synthesized elsewhere and then targeted to mitochondria or retained in mitochondrial membranes. Of note, plasmanylcholines cannot be directly converted to plasmenylcholine (17). Their metabolism requires therefore their conversion by removal of the (phospho)choline group and/or their deacylation. As postulated above for phosphatidylcholine, the phospholipase A2 that carries out the latter reaction might not work if the fatty acyl group is ethylbranched.

### Increase of methyl-branched FAs in ECHDC1 deficiency is lower than expected in most analyzed tissues

ECHDC1 can decarboxylate ethylmalonyl-CoA and, with an about 20-fold lower catalytic efficiency, methylmalonyl-CoA (6). Its postulated function is to destroy these two compounds, which otherwise might replace malonyl-CoA in the synthesis of FAs and thereby cause the formation of ethyl- or methyl-branched FAs (2). Previous data obtained on adipocyte models (3T3-L1 and L929) indicated that knocking out of ECHDC1 led indeed to a 2- to 5-fold increase in the formation of methyl-branched FAs. In contrast, evidence for the formation of ethyl-branched FAs in cell lines was only obtained by feeding the cells with stable isotopically labeled ethylmalonate.

These findings motivated us to generate a mouse model deficient in ECHDC1 in order to see whether tissues accumulated branched FAs *in vivo* and if this had toxic consequences for the metabolism of FAs. Curiously, we obtained clear evidence for the accumulation of ethyl-branched fatty acids in ECHDC1 knockout mice, whereas increases in methyl-branched were subtle or absent. An explanation for this is that propionyl-CoA levels in the cytosol are much lower in mice than in cell models. This is supported by the fact that mouse adipose tissue in comparison to adipocyte cell lines contains about 10-fold less straight-chain FAs with an uneven carbon number (*i.e.*, C15:0 and C17:0), which are mainly synthesized by elongation of propionyl-CoA instead of the classical acetyl-CoA by FASN (18). To a certain extent this is likely a consequence of the relative vitamin B12 deficiency observed in cultured cell lines, which impedes mitochondrial methylmalonyl-CoA and propionyl-CoA metabolism. This is supported by the observation that vitamin B12 supplementation to cells decreased the formation of uneven and methyl-branched FAs, derived from propionyl-CoA incorporation (uneven FAs) or methylmalonyl-CoA incorporation (methyl-branched FAs), respectively (1, 2). An exception to this observation are the intraorbital glands (comprising both Harderian gland and intraorbital lacrimal gland), where methyl-branched FAs are increased despite low levels of FAs with uneven carbon numbers (data not shown). Of note, it has previously been shown that Harderian glands of guinea pigs produce methyl-branched-chain FAs under normal conditions (19). At present, it remains unclear how and why these glands tend to make more methyl-branched FAs.

### Increase of ethylmalonyl-CoA along with the appearance of 2,2-dimethylmalonyl-CoA and subcellular localization of ECHDC1

As expected, the loss of ECHDC1 leads to a several-fold increase of ethylmalonate in urine and in tissues (the kidney and liver, data not shown). Unlike the effect on ethyl-branched FAs, this increase is not an all or none response (Fig. 6*C*). Indeed, ethylmalonate is present in wild-type mice, most likely due to the metabolism of L-alloisoleucine, a noncanonical amino acid formed as a side product during the transamination of L-isoleucine (20, 21).

To our surprise, we found that an isomer of ethylmalonate, identified as 2,2-dimethylmalonate, was highly increased in ECHDC1 knockout mouse urine (Fig. 6*I*) and tissues (data not shown). Similarly, 2,2-dimethylmalonyl-CoA accumulated in ECHDC1 knockout liver, especially in the mitochondria (Fig. 6, *G* and *K*). Though this has not been investigated, these findings suggest that ECHDC1 also acts on 2,2-dimethylmalonyl-CoA. This could indicate that ethylmalonyl-CoA is mostly a side product of the cytosolic ACC acting on butyryl-CoA, while 2,2-dimethylmalonyl-CoA is mostly a side product of the mitochondrial propionyl-CoA carboxylase (PCC) or the mitochondrial ACC isoform acting on isobutyryl-CoA, a product of valine degradation (22) (Fig. 6*L*). Previous measurements of specific enzymatic activity in subcellular fractionation of rat liver (6) and published proteomic data (23) support the notion that small amount of mitochondrial ECHDC1 is localized in mitochondria and thereby might help to prevent accumulation of 2,2-dimethylmalony-CoA (Fig. 6*L*). Of note, the overall activity of mitochondrial ECHDC1 must be quite limited, given that in ACADS (24) and ETHE1 (25) deficiencies ethylmalonate and likely mitochondrial ethylmalonyl-CoA accumulate even though ECHDC1 is present.

Accumulating 2,2-dimethylmalonyl-CoA, similarly to methylmalonyl-CoA or ethylmalonyl-CoA, might replace malonyl-CoA in FA elongation processes. If this is the case, only the first two steps of elongation are biochemically possible (*i.e.*, ketoacyl-synthesis and ketoacyl reduction) because there is no room to create a double bond between C_2_ and C_3_. This prevents further chain extension and might thereby reduce products of mitochondrial FA synthesis such as lipoate and acyl-carrier-protein-coupled lipids that play a role in the assembly of respiratory chain complexes, iron sulfur complexes, and the mitochondrial ribosomes (26, 27, 28). While the functional relevance of this is still unclear, the observation of increased 2,2-dimethylmalonyl-CoA in ECHDC1 knockout mitochondria underlines that the broad substrate spectrum of ECHDC1 allows cells to limit the accumulation of a series of metabolic side products that can disturb cellular FA metabolism.

Based on our observations in ECHDC1 knockout mice, we expect that individuals with complete ECHDC1 deficiency (*i.e.*, with two mutated alleles) will show an increase in urinary excretion of ethylmalonate, 2,2-dimethylmalonate, and branched-chain acylglycines (see below). Of note, Fogh and colleagues have very recently reported three patients with elevated urinary ethylmalonate excretion that harbored two variants in *ACADS* together with a single pathogenic variant in *ECHDC1* (29). Deficiency in ACADS is expected to lead to increased butyryl-CoA, which serves as a substrate for the formation of ethylmalonyl-CoA. Thus, a heterozygous deficiency in ECHDC1 (*i.e.*, enzyme that destroys ethylmalonyl-CoA) might suffice to increase urinary secretion of ethylmalonic acid. Future studies will reveal whether biallelic inactivation of *ECHDC1* might lead to a human inborn error of metabolism.

### Presence and origin of medium chain (ethyl-)branched fatty acid derivatives in urine

The most impressive evidence for the synthesis of branched FAs was that ECHDC1 knockout mice excrete a multitude of ethyl-branched medium-chain FAs (*i.e.*, 8–12 carbons) as glycine conjugates in the urine. The relative increase in their concentration is usually more than 20-fold (Table 1). Their identification as ethyl-branched FAs could be ascertained for some of them by comparison with spike-in standards (Figs. 5*A* and S5*A*) or by demonstration that labeled ethylmalonate was used as a precursor (Figs. 5, *C* and *D* and S5, *C* and *D*). These molecules correspond to acyl compounds that are either saturated or at various stages of oxidation (*i.e.*, unsaturated; presence of a hydroxyl group, a keto group, or a second carboxylic group). Based on the concentrations of the (putative) 4-ethylhexanoylglycine (Fig. 5*A*, peak II) in urine and a urinary creatinine excretion of 400 μg/day, we estimate that ECHDC1-deficient mice excrete approximately 50 nmol of 4-ethylhexanoylglycine per day. Given that branched-chain FAs represent only a very small part of the total FA pool, it is conceivable that these compounds are remainders of an inefficient degradation of ethyl-branched fatty acids in mitochondria.

Three arguments indicate that a large portion of ethyl-branched glycine adducts might be the result of abortive fatty acid synthesis by FA synthase. First, in experiments with purified FASN, we had previously noted that this enzyme not only forms long-chain ethyl-branched FAs but also medium-chain ethyl-branched FAs in quantities that are roughly the same as the corresponding straight chains isomers (see C8:0 and C10:0 FAs, Fig. S4 in previous paper (2)). This suggests that the synthesis of the ethyl-branched FAs often aborts before the chain reaches its normal end point (16 carbons), possibly because the synthesis of such species is extremely slow, leaving more chance to the thioesterase domain of FASN to act “prematurely.” Second, when we fed mice with deuterium labeled ethylmalonic acid, we observed deuterium incorporation in acylglycines within 4 h after gavage. Lastly, glycine adducts were relatively more abundant in total liver than in isolated mitochondria (data not shown). Given that classical FA synthesis is a cytosolic process, while FA degradation is largely a mitochondrial process, the lack of mitochondrial enrichment might point toward a problem during synthesis.

This being said, we cannot discount the possibility that degradation of branched-chain FAs might be contributed by peroxisomes (at least partially), while the enzyme GLYAT (glycine acyl-transferase) is largely sublocalized in mitochondria (30). Another possible source of branched FAs is mitochondrial FA synthesis. Thus, we cannot exclude that some of the oxidized isomers of medium-chain FA compounds excreted by ECHDC1 knockout mice are 2,2-dimethylated on C_2_ with a keto- or a hydroxy-function on C_3_, following an abortive 2,2-dimethylmalonyl-CoA incorporation.

To the best of our knowledge, the beta-oxidation of ethyl-branched FAs has not been studied, yet several pathways might be involved in the degradation of ethyl-branched FAs. One possibility is that long-chain ethyl-branched FAs (observed only in saturated forms, mostly in BAT) are first degraded by beta-oxidation until they reach the size of medium-chain ethyl-branched FAs. The failure to further metabolize these medium-chain ethyl-branched CoAs in mitochondria might then lead to their accumulation and the formation of glycine adducts. Metabolism of the ethyl-branched 2-ethylhexanoate (a plasticizer) has been investigated in several studies in humans and in rats. This compound is partially metabolized by beta-oxidation and by omega-oxidation, indicating that the presence of an ethyl branch slows down or may even prevent normal beta oxidation (8, 31, 32). Thus, the presence of ethyl-branched FAs in different oxidation states (Figs. 5, *E–G*, S5, *E–G*, and S6), might be taken as an indication that partial processing by beta-oxidation and/or omega-oxidation occurs and that the liver conjugates these species to glycine and taurine, before urine excretion. However, comparable reaction intermediates might also occur during FA synthesis.

Importantly, some ethyl-branched FAs might be difficult to metabolize or to remove from complex lipids such as phosphatidylcholine or plasmanylcholine species. Thus, some of them might accumulate with age leading to detrimental consequences that are likely to be most apparent in long-lived species. Of note, recent studies have indicated that ECHDC1 might play a role in the development of breast cancer (33, 34). Given that the mammary epithelium is in close vicinity to adipocytes, it is tempting to speculate that abnormal FA metabolites released from adipocytes might play a role in mammary epithelium transformation.

## Experimental procedures

### Materials

3-pyridinemethanol, potassium tert-butoxide, toluene, dichloromethane, ethylmalonic acid, 2,2-dimethylmalonic acid, methylsuccinic acid, glutaric acid, n-adipic acid, n-suberic acid, n-sebacic acid, n-octanoylglycine, n-decanoylglycine, glycine methylester hydrochloride, 2-ethylhexanoic acid, 2-ethyloctanoic acid, 4-ethyloctanoic acid, tetrahydrofurane, carbonyldiimidazole, 2-mercapto-ethanol, 2-(N-morpholino) ethanesulfonic acid (MES) monohydrate, and coenzyme A were from Sigma-Aldrich (now Merck). Ethylmalonic acid (methyl-d3) was from Cambridge Isotope Laboratories. Methanol, acetonitrile, isopropanol (MS-grade), formic acid, and ammonium formate (all MS-grade) were from Biosolve. n-Hexane was from VWR chemicals. Ethylmalonyl-CoA was from CoALA Biosciences. Hydrochloric acid (37%) was from Merck. 1-ethyl-3-(3-dimethylaminopropyl)-carbodiimide hydrochloride (EDAC) was from Thermo Fisher Scientific.

### Generation of ECHDC1 deficient mice

Animal procedures were approved by the local animal ethics commission (BCHM-2021-001). ECHDC1 KO mice were created using the CRISPR/Cas9 genome editing system as previously described (35, 36). Plasmids were prepared by ligating annealed pairs of primers shown below into the vector pX330-U6-Chimeric_BBCBh-hSpCas9 (a gift from F. Zhang, Massachusetts Institute of Technology; Addgene plasmid no. 42230). ECHDC1 was targeted in exon 5 before a highly conserved glutamate residue in the motif GGGAEFTT by two different guide RNAs (targets 4 and 5). Pairs of primers to generate a construct for guide RNA 4 were 5′-CAC CGG ACC GTT AAT AAG TGT TGC TC(s) and 5′-AAA CGA GCA ACA CTT ATT AAC GGT CC (as). Pairs of primers to generate a construct for guide RNA 5 were 5′-CAC CGG CTG GTT CAA GGC TGG GCA AT(s) and 5′-AAA CAT TGC CCA GCC TTG AAC CAG CC (as). All constructs were validated by sequencing. These guide RNAs were chosen in a larger selection of single-guide RNAs based on their superior capacity to cleave the target site of the ECHDC1 cloned in the pCAG-EGxxFP vector (gift from Ikawa Mashiko; Addgene plasmid number 50716) (37). Both plasmids were comicroinjected into the male pronucleus of one-cell-stage embryos derived from C57BL/6N mice. Genomic DNA from mouse tails was extracted and used to PCR-amplify the region that encompasses the mutated sequence. PCR products were analyzed by Sanger sequencing (Genewiz). A deletion of ten nucleotides in exon 5 was obtained in the heterozygous state in mice. The result of this mutation is a frameshift and a premature stop codon at the beginning of exon 6 (Fig. 1*B*). Homozygous knockout mice were compared to wild-type and heterozygous littermates.

### Mouse experiments

Mice used for experiments were deeply anesthetized by intraperitoneal injection with ketamine and xylazine at 100 mg/kg and 12.5 mg/kg, respectively, as recommended by the International Institutional Animal Care and Use Committee (IACUC) of the American Association for Laboratory Animal Science (AALAS) guidelines (38).

Tissues were fixed with 4 % formaldehyde overnight, dehydrated, and embedded in paraffin. A histopathological analysis was performed on hematoxylin-eosin-stained slides from the following tissues: liver (6 +/− and 6 −/−), spleen (4 +/− and 4 −/−), heart (10 +/− and 10 −/−), lung (2 +/− and 2 −/−), thymus (2 +/− and 2 −/−), kidney (10 +/− and 10 −/−), brain and cerebellum (7 +/− and 7 −/−), adrenal (2 +/− and 2 −/−), skin (4 +/− and 4 −/−), testis/uterus and ovaries (2 +/− and 2 −/−), brown fat (4+/− and 4 −/−), white fat (4+/− and 4 −/−), perianal gland (2+/− and 2 −/−), quadriceps (4+/− and 4 −/−), pancreas (2+/− and 2 −/−), and spinal cord (4+/− and 4 −/−). Frozen sections of the liver, kidney, heart as well as white and brown adipose tissue (BAT) from 9-month-old mice (3 +/− and 3 −/−) were stained with Oil red O to compare neutral lipids. These organs were flash frozen on dry ice in Optimal Cutting Temperature Control (OCT) medium (TissueTek) to embed tissues before frozen sectioning using a cryostat.

To isolate mitochondria we followed a method described by Frezza *et al.* (39). Briefly, fresh livers from overnight-fasted wild-type and ECHDC1-deficient mice were removed, rinsed, minced, and homogenized using a Potter homogenizer in ice-cold isolation buffer pH 7.4 (10 mM Tris/MOPS, 1 mM EGTA/Tris, and 200 mM sucrose). Cellular debris and nuclei were pelleted by centrifugation for 10 min at 600*g* and 4 °C. The supernatants were centrifuged for 10 min at 7000*g* and 4 °C. The resulting supernatants containing the microsomal fraction were kept, and the pellets containing mitochondria-enriched fraction were washed once with ice-cold isolation buffer and centrifuged again for 10 min at 7000*g* and 4 °C. Pellets were resuspended in the residual small volume of isolation buffer, and protein concentrations were measured using the Pierce bicinchoninic acid (BCA) protein assay kit (Thermo Fischer Scientific) before freezing at –80 °C in 50 μl aliquots. In order to isolate microsomes (35) from the supernatants, an ultracentrifugation for 60 min at 80,000*g* and 4 °C was performed and the pellets were resuspended in fresh isolation buffer. After a second ultracentrifugation at 80,000*g* for 60 min at 4 °C, the pellets were resuspended in a small amount of residual buffer. Protein concentrations were determined before freezing at –80 °C in 50 μl aliquots.

For metabolomic and lipidomic analyses, tissues were snap-frozen in liquid nitrogen immediately after dissection. In total, 500 μl of cold methanol and 350 μl of cold water were added to ≈25 mg of frozen tissue in 2 ml tubes containing small ceramic beads (≈8 of 1.4 and ≈3 of 2.9 mm diameter) kept on ice. Samples were homogenized with three cycles of 20 s at 10,000 rpm using a Precellys evolution tissue homogenizer (Bertin) connected to a Cryolysis cooling system (Bertin). One milliliter of cold chloroform was added to perform a Folch extraction in a second round of homogenization (40). Homogenized samples were centrifuged for 10 min at 16,000*g* and 4 °C. The upper layer (aqueous fraction) and the lower layer (organic fraction) were collected in separate tubes and stored at –80 °C until analysis. Metabolite extractions from mitochondria and microsomes were performed as described above (Folch extraction) using an amount that corresponded to 5 mg of mitochondrial or microsomal proteins.

Urine was obtained from mice either by collecting drops resulting from spontaneous urination of living mice or by aspiration of the bladder in freshly euthanized mice. Creatinine was assessed by Jaffe reaction (41). The volume of urine containing 0.05 μmol of creatinine (= 5.656 μg) was added to methanol in order to reach a final volume of 100 μl in 1.5 ml reaction tubes. Samples were mixed and frozen at –20 °C at least overnight. Then, the samples were centrifuged (16,000*g* during 10 min at 4 °C) and 50 μl of the supernatants was transferred in vials for LC-MS analysis. To trace the fate of ethyl-branched FAs, ECHDC1 heterozygote and ECHDC1 deficient mice were gavaged in the morning with 30 mg of (methyl-d3)-ethylmalonic acid in 100 μl of 0.9% NaCl. Urine was collected 4 h later and kept frozen until analysis by LC-MS.

### Synthesis of 2-ethylhexanoylglycine, 2-ethyloctanoylglycine, and 4-ethyloctanoylglycine

Fresh solutions of EDAC, glycine methyl ester, and 2-ethylhexanoic acid, 2-ethyloctanoic acid, or 4-ethyloctanoic acid were prepared in 100 mM MES buffer, pH 5.5. We incubated 20 mM EDAC, 20 mM methylglycine together with 20 mM ethyl-branched FA solutions in 100 mM MES buffer pH 5.5 at room temperature for 2 h in a final volume of 50 μl. The reaction was stopped by addition of 2-mercaptoethanol (1 μl). After alkalinization with a few μL of 2 M NaOH to a pH between 10 and 11, samples were incubated for 30 min at 70 °C to remove the methyl groups. The resulting products were diluted in water and used as standards for LC-MS analyses.

### Synthesis of 2,2-dimethylmalonyl-CoA

Coenzyme A esters of ethylmalonic acid (Sigma; 102687) and 2,2-dimethylmalonic acid (Sigma; D168009) were synthesized in two steps as described in (42). First, the carboxylic acids were activated by carbonyldiimidazole (CDI) (Alfa Aesar; A14688). For each reaction, 4.2 mg (26 μmoles) CDI was dissolved in 200 μl tetrahydrofurane (Sigma; 401757). The carboxylic acid (31 μmoles) was added and the reaction was incubated in a shaker at 200 rpm and 22 °C for 1 h. In the second step, the resulting CDI-activated intermediate was esterified with free Coenzyme A (Sigma; C4282). Coenzyme A (6.4 μmoles) was dissolved in 50 μl of 0.5 M NaHCO_3_ and added to the reaction mixture, which was incubated for an additional hour at 22 °C with shaking at 200 rpm, flash frozen in liquid nitrogen, and dried down under a stream of nitrogen. The samples were resuspended in 1 ml water.

### GC-MS analysis of FAs

GC-MS analysis of FA methyl esters was performed using an Agilent 7890A apparatus equipped with a 30 m DB-5 ultra-inert capillary column and connected to an Agilent 5977 mass detector acquiring in combined Selected-Ion Monitoring (SIM) and scanning mode as described in (2). Methyl ester and 3-pyridylcarbinol (“picolinyl”) ester transesterifications of FAs were performed as described in (2).

### GC-MS analysis of organic acids in urine

Organic acids in urine were measured in a routine workflow of a clinical Biochemical Genetics laboratory (Department of Laboratory Medicine, Cliniques universitaires St Luc, UCLouvain, Brussels, Belgium). Briefly, urine was acidified by addition of hydrochloric acid and was extracted three times with ethyl acetate. The pooled organic phase was dried down under nitrogen flow, and organic acids were trimethylsilylated and analyzed by gas chromatography–mass spectrometry (ThermoScientific, Trace 1310) using an Agilent CP-Sil 8 CB-MS 30 m x 0.25 mm x 0.25 μm capillary column (43).

### LC-MS analysis of hydrophilic metabolites

The upper aqueous layer of the liquid–liquid extraction (Folch extraction) of tissues was dried down using a SpeedVac vacuum concentrator (Thermo Scientific), then resuspended in 50% methanol in water. The resulting solution was again centrifuged for 10 min at 4 °C and 16,000*g*. Subsequently, the clear supernatant was transferred to a fresh vial before analysis by LC-MS.

Experiments on aqueous fractions from tissues (including urine methanolic extracts) used the same method described for metabolomic analysis (CoA esters) in cells (2). LC-MS analysis of aqueous samples extracted from cells, urine, or from aqueous fractions obtained by Folch extraction of tissues was performed using a LC-MS qTOF scanning m/z between 69 and 1700 as described in Veiga da Cunha *et al*. (35). Briefly, 5 μl was injected and subjected to ion pairing chromatography with an Inertsil 3 μm particle ODS-4 column (150 × 2.1 mm, GL Biosciences) on an Agilent 1290 HPLC system using hexylamine as pairing agent.

### LC-MS analysis of hydrophobic metabolites

Prior to LC-MS analysis, 200 μl of the organic layer obtained from the liquid–liquid extraction according to Folch was dried down under nitrogen, then resuspended in 100 μl of methanol: isopropanol (1:1 v:v). After centrifugation for 10 min at 4 °C and 16,000*g*, the supernatant was transferred into fresh vials. The chromatography method was adapted from (44). Briefly, 5 μl of the extract was injected and subjected to reverse-phase liquid chromatography using a C30 column (150 × 2 mm, particle size 2.6 μm, pore diameter: 150 Å, Thermo Fisher Scientific) on an Agilent 1290 HPLC system. The mobile phases A (acetonitrile: water 60:40 v/v, 10 mM ammonium formate and 0.1 % formic acid) and B (isopropanol:acetonitrile:water 90:10:1 v/v/v, 10 mM ammonium formate and 0.1 % formic acid) were used at 30 °C with a flow rate of 0.2 ml/min, using the following concentrations and linear gradients: 0–3 min: 30% B; increase of B to 43% over 5 min; increase of B to 50% over 1 min; increase of B to 90% over 9 min; increase of B to 99% over 8 min; 99% B from 26 to 30 min; 30% B from 30 to 35 min. Samples were injected twice, with the electrospray ionization source being operated once in negative and once in positive mode for analysis with an Agilent 6550 ion funnel mass spectrometer. The ESI spray voltage was 3500 V, sheath gas 350 °C at 11 l/min, nebulizer pressure 35 psig, and drying gas 200 °C at 14 l/min. An m/z range from 70 to 1700 was acquired with a frequency of 1 spectrum per second.

### Untargeted analysis of LC-MS data

Peaks were aligned and integrated and their abundance was compared between controls and ECHDC1 knockout samples using MassHunter Mass Profiler software (Agilent) in order to determine the features that were significantly higher or lower between the two groups. Extracted ion chromatograms of the most significant metabolites (>4-fold increase or decrease) were validated and peaks were integrated manually. Compounds were identified based on exact mass and elution times.

### Statistical analysis

Data were analyzed with GraphPad Prism 9.0. When multiple groups were analyzed, a one-way ANOVA was followed by post-hoc testing corrected according to Šidák (45).

## Supporting information

This article contains supporting information (2).

## Conflict of interest

The authors declare that they have no conflicts of interest with the contents of this article.
