# Supplementary material for: ECHDC1 knockout mice accumulate ethyl-branched lipids and excrete abnormal intermediates of branched-chain fatty acid metabolism
Source: J Biol Chem. 2021 Aug 19;297(4):101083. doi: 10.1016/j.jbc.2021.101083 (PMC8473548; doi:10.1016/j.jbc.2021.101083)
Supplement: Figures S1–S7 [file mmc1.pdf]

SUPPLEMENTARY DATA

ECHDC1 knock-out mice accumulate ethyl-branched lipids in tissues and excrete abnormal intermediates of branched-chain fatty acid metabolism

Joseph P. Dewulf[1,2,4], Stephanie Paquay[1,4], Etienne Marbaix[5,6], Younes Achouri[7], Emile Van Schaftingen[1], Guido T. Bommer[1]

[1] Department of Biochemistry, de Duve Institute, UCLouvain, Brussels, Belgium; [2] Walloon Excellence in Lifesciences and Biotechnology (WELBIO), Brussels, Belgium
[3] Department of Laboratory Medicine, University Hospital St. Luc, UCLouvain, Bruxelles, Belgium
[4] Department of Neuropediatrics, University Hospital St. Luc, UCLouvain, Bruxelles, Belgium
[5] Department of Anatomical Pathology, University Hospital St. Luc, UCLouvain, Bruxelles, Belgium
[6] Department of Cell Biology, de Duve Institute, UCLouvain, Bruxelles, Belgium
[7] Transgenesis Platform, de Duve Institute, UCLouvain, Bruxelles, Belgium

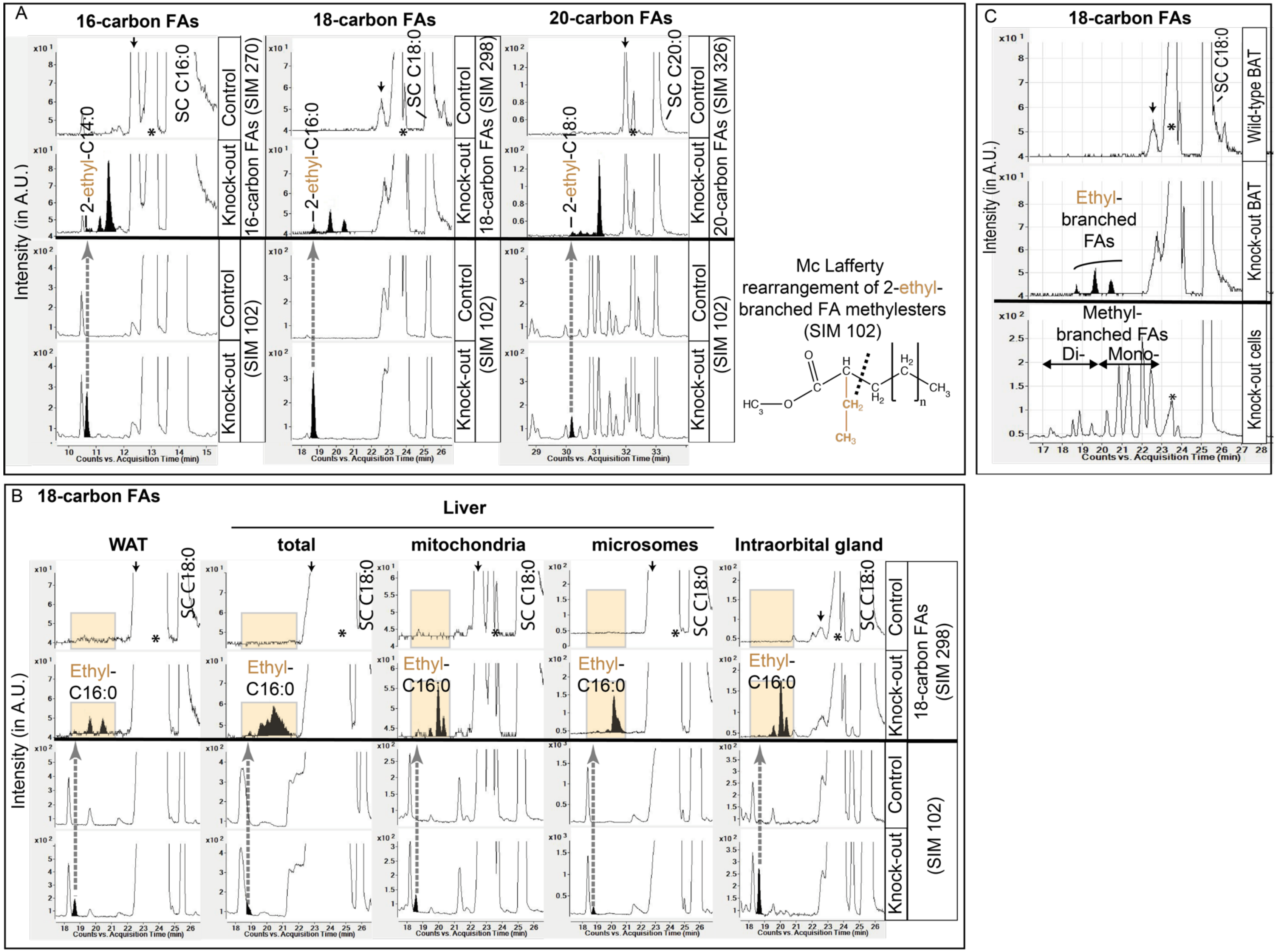
A
16-carbon FAs
18-carbon FAs
20-carbon FAs
Intensity (in A.U.)
SC C16:0
SC C18:0
SC C20:0
2-ethyl-C14:0
2-ethyl-C16:0
2-ethyl-C18:0
Control
Knock-out
16-carbon FAs (SIM 270)
18-carbon FAs (SIM 298)
20-carbon FAs (SIM 326)
(SIM 102)
Counts vs. Acquisition Time (min)
Mc Lafferty rearrangement of 2-ethyl-branched FA methylesters (SIM 102)
B 18-carbon FAs
Liver
WAT
total
mitochondria
microsomes
Intraorbital gland
Ethyl-C16:0
18-carbon FAs (SIM 298)
C
18-carbon FAs
SC C18:0
Wild-type BAT
Knock-out BAT
Knock-out cells
Ethyl-branched FAs
Methyl-branched FAs
Di-
Mono-

FIGURE S1. 2-ethyl-branched FA identification and detection of ethyl-branched FAs in additional tissues.
A. GC-MS elution profiles of 16-, 18- and 20-carbon FAs (SIM 270, 298 and 326, respectively) in brown adipose tissue showing co-elution of the putative 2-ethyl-branched FAs (upper two panels) with the m/z 102 McLafferty rearrangement fragment characteristic for 2-ethyl-branched FA methylesters (lower two panels). **B**. GC-MS elution profiles of 18-carbon FAs in WAT, liver (total, mitochondria and microsomes) and intraorbital glands showing co-elution of the between putative 2-ethyl-branched FAs and the m/z 102 fragment. **C**. GC-MS elution profile comparison between ethyl-branched FAs observed in brown adipose tissue and methyl-branched FAs observed in ECHDC1 knock-out adipocytes (same sample as Fig. S5i in Dewulf *et al.* 2019). For abbreviations, see Fig. 1.

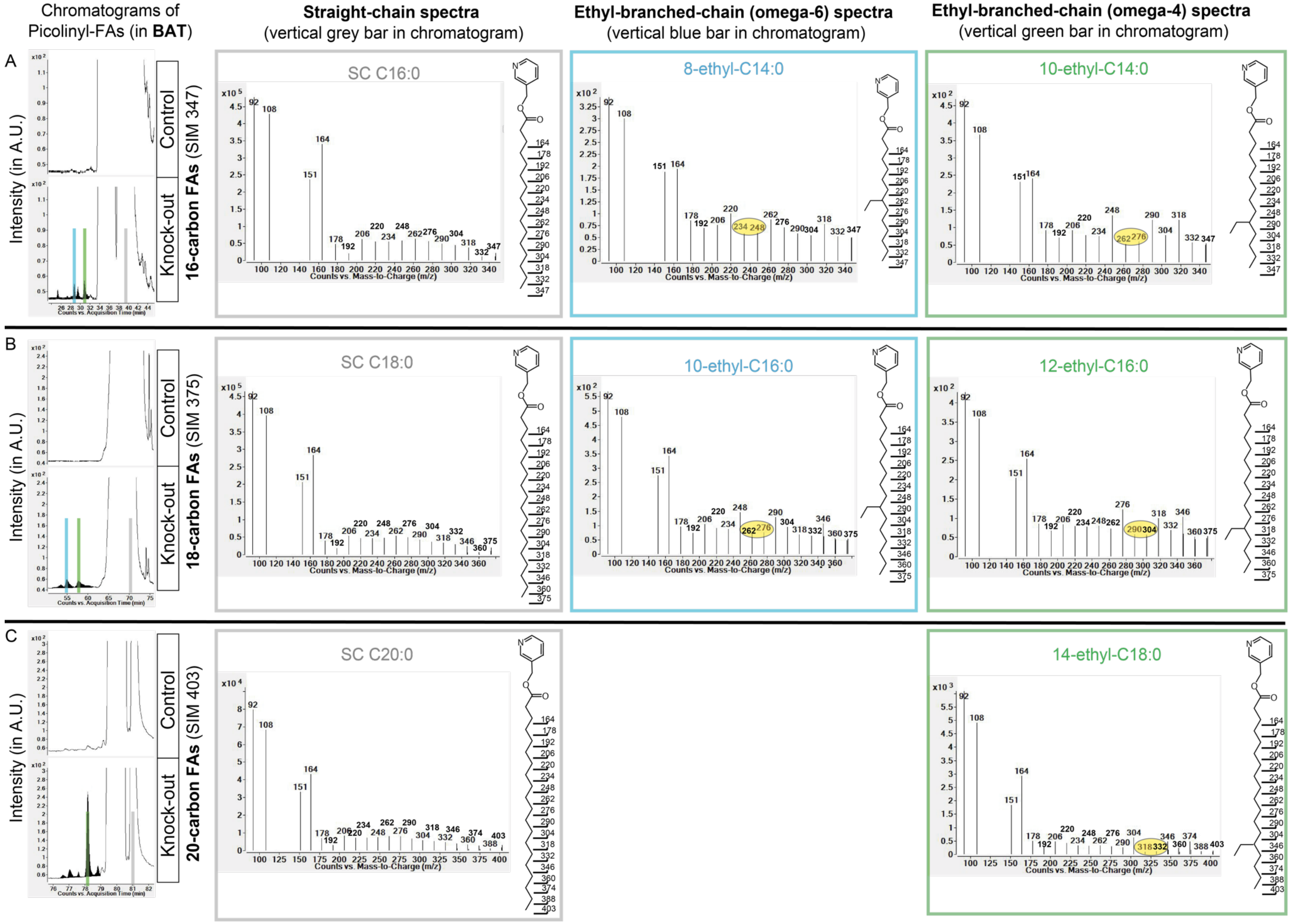

Chromatograms of Picolinyl-FAs (in BAT)
Straight-chain spectra (vertical grey bar in chromatogram)
Ethyl-branched-chain (omega-6) spectra (vertical blue bar in chromatogram)
Ethyl-branched-chain (omega-4) spectra (vertical green bar in chromatogram)
A
B
C
Intensity (in A.U.)
Control
Knock-out
16-carbon FAs (SIM 347)
18-carbon FAs (SIM 375)
20-carbon FAs (SIM 403)
SC C16:0
8-ethyl-C14:0
10-ethyl-C14:0
SC C18:0
10-ethyl-C16:0
12-ethyl-C16:0
SC C20:0
14-ethyl-C18:0
Counts vs. Acquisition Time (min)
Counts vs. Mass-to-Charge (m/z)

FIGURE S2. Putative identification of ethyl-branched FAs by GC-MS analysis after 3-pyridyl-carbinol ("picolinyl") derivatization of samples from brown adipose tissue.

Analysis of (**A**) 16-carbon fatty acids (SIM 347), (**B**) 18-carbon fatty acids (SIM 375) and (**C**) 20-carbon fatty acids (SIM 403). In each case, the leftmost panel shows the elution profile from the column for a control (upper panel) and an ECHDC1-KO sample (lower panel). Ethyl-branched FAs peaks (filled in black) are only detected in ECHDC1 KO samples. The panels to the right show spectra corresponding to the straight chain (SC) species and to the ethylbranched species highlighted in blue or in green in the leftmost panel. The diminished intensity of neighbouring peaks (highlighted in yellow) indicates the presence of ethyl branches , in comparison to the straight-chain FA spectrum (in grey). The masses of these paired peaks indicate that the predominant branching sites are ω-6 (in blue) and ω-4 (in green). Corresponding chemical structures are represented on the right of the spectra.

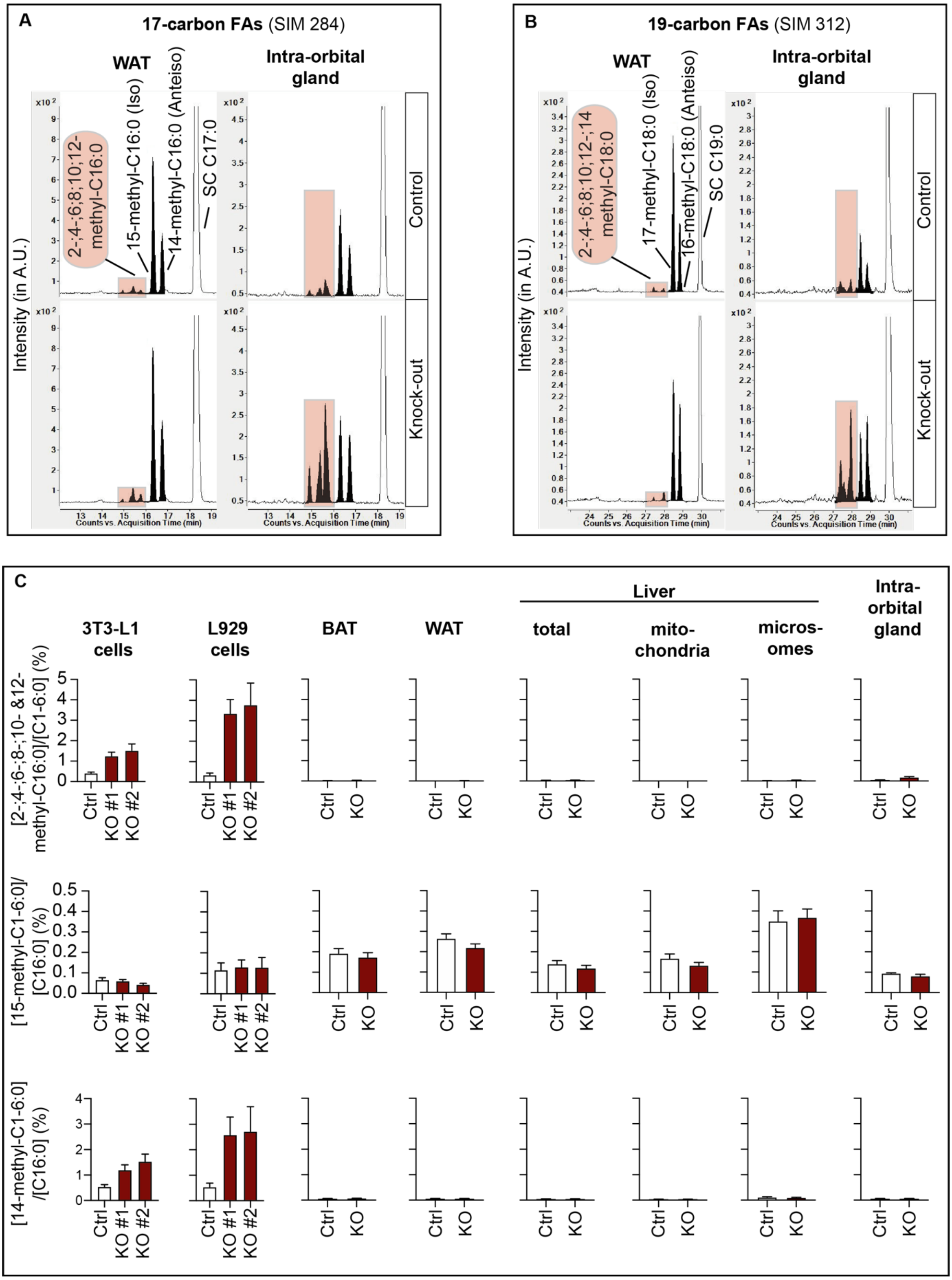
A
17-carbon FAs (SIM 284)
WAT
Intra-orbital gland
2-;4-;6;8;10;12-methyl-C16:0
15-methyl-C16:0 (Iso)
14-methyl-C16:0 (Anteiso)
SC C17:0
Intensity (in A.U.)
Control
Knock-out
Counts vs. Acquisition Time (min)
B
19-carbon FAs (SIM 312)
WAT
Intra-orbital gland
2-;4-;6;8;10;12-;14 methyl-C18:0
17-methyl-C18:0 (Iso)
16-methyl-C18:0 (Anteiso)
SC C19:0
Intensity (in A.U.)
Control
Knock-out
Counts vs. Acquisition Time (min)
C
3T3-L1 cells
L929 cells
BAT
WAT
Liver
total
mito-chondria
micros-omes
Intra-orbital gland
[2-;4-;6-;8-;10- &12-methyl-C16:0]/[C1-6:0] (%)
[15-methyl-C1-6:0]/[C16:0] (%)
[14-methyl-C1-6:0]/[C16:0] (%)
Ctrl
KO #1
KO #2
KO

FIGURE S3. Analysis of additional methyl-branched fatty acids.
(See also Fig. 3)
A. GC-MS elution profiles of 17-carbon FAs (SIM 284) in white adipose tissue and intraorbital glands from control and ECHDC1 knock-out mice. 2-,4-, 6-, 8-, 10- and 12-methyl-branched FAs are highlighted in light red and were identified as described in Dewulf et al. (2019).
B. GC-MS elution profiles of 19-carbon FAs (SIM 312) between control and ECHDC1 knock-out samples in white adipose tissue and intraorbital glands. 2-,4-, 6-, 8-, 10-, 12- and 14-methyl-branched FAs are highlighted in light red.
C. Quantification of the indicated methyl-branched fatty acids in the indicated organs by GC-MS. Values are means +/- SEM of at least 6 mice (except for intraorbital glands where n= 3), and are represented relative to the abundance of the corresponding non-methylated fatty acid. Note that the data for cell lines is taken from our previous work to facilitate the comparison with the mouse samples (Dewulf et al. 2019).
SC, Straight Chain; BAT, Brown Adipose Tissue; WAT, White Adipose Tissue; A.U. Arbitrary Units.

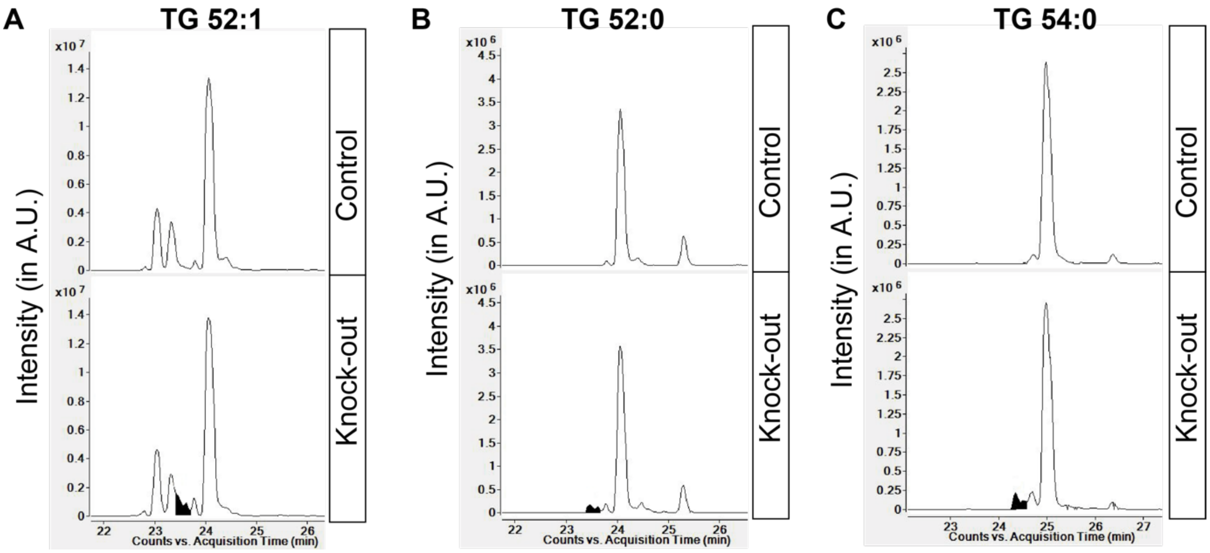

FIGURE S4. Likely ethyl-branched FA-containing triglycerides detected by LC-MS analysis in brown adipose tissue.

A. Elution profile of TG 52:1 species ($C_{55}H_{104}O_6$, *[M+NH4]+ 878.8171*).

B. Elution profile of TG 52:0 species ($C_{55}H_{106}O_6$, *[M+ NH4]+ 880.8328*).

C. Elution profile of TG 54:0 species ($C_{57}H_{110}O_6$, *[M+ NH4]+ 908.8641*).

Likely ethyl-branched FA-containing species are filled in black. A.U., Arbitrary Units

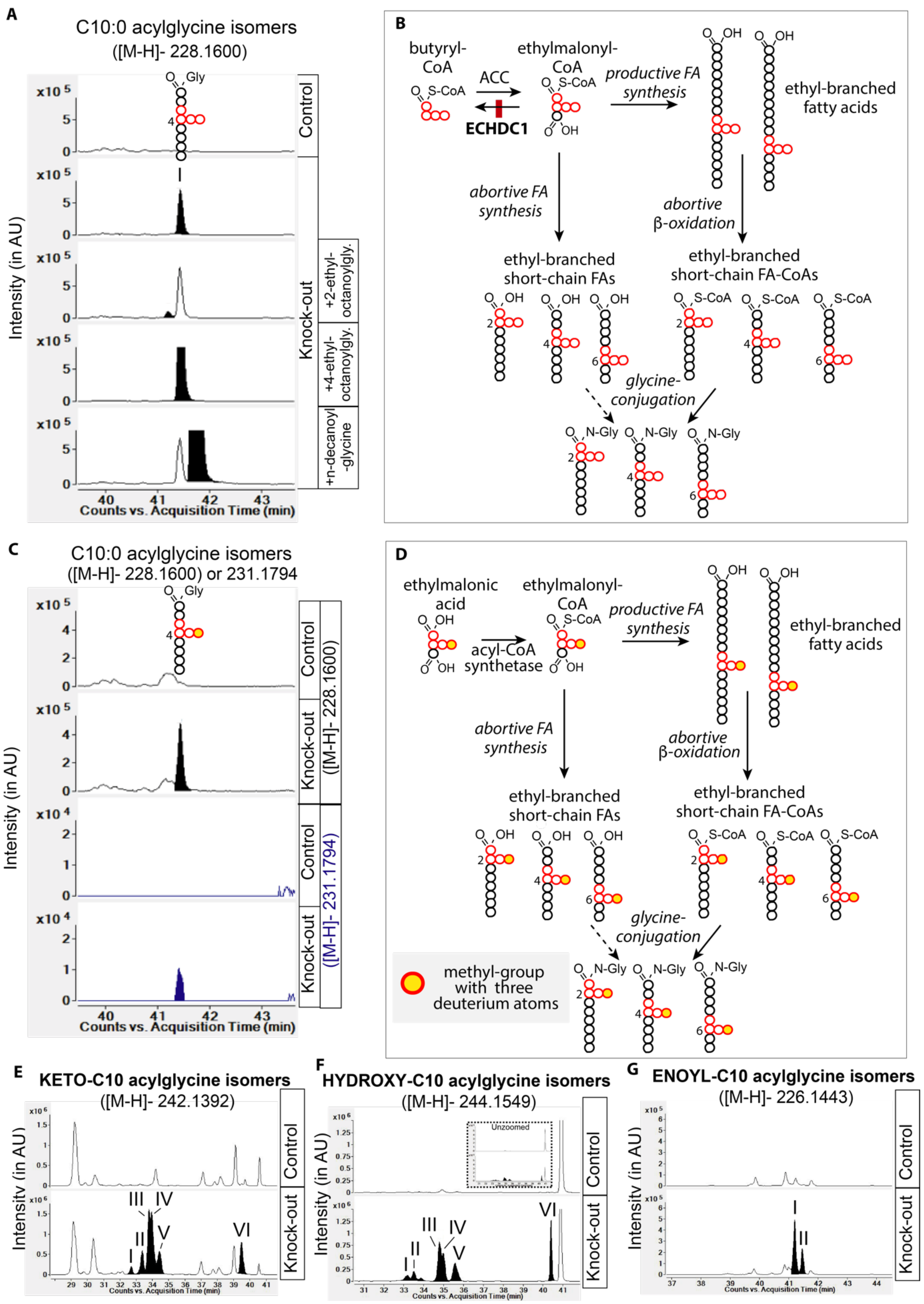

A
C10:0 acylglycine isomers
([M-H]- 228.1600)
Intensity (in AU)
Control
Knock-out
+2-ethyl-octanoylgly.
+4-ethyl-octanoylgly.
+n-decanoyl-glycine
Counts vs. Acquisition Time (min)
B
butyryl-CoA
ACC
ECHDC1
ethylmalonyl-CoA
productive FA synthesis
ethyl-branched fatty acids
abortive FA synthesis
abortive β-oxidation
ethyl-branched short-chain FAs
ethyl-branched short-chain FA-CoAs
glycine-conjugation
C
C10:0 acylglycine isomers
([M-H]- 228.1600) or 231.1794
([M-H]- 228.1600)
([M-H]- 231.1794)
D
ethylmalonic acid
acyl-CoA synthetase
methyl-group with three deuterium atoms
E
KETO-C10 acylglycine isomers
([M-H]- 242.1392)
F
HYDROXY-C10 acylglycine isomers
([M-H]- 244.1549)
Unzoomed
G
ENOYL-C10 acylglycine isomers
([M-H]- 226.1443)

FIGURE S5. 4-Ethyl-branched octanoylglycine and derivatives accumulate in urine from ECHDC1-deficient mice.
A. Extracted ion chromatogram (EIC) of 10-carbon acylglycine isomers in urine and spike-in experiments allowing the identification of peak I as 4-ethyl-octanoylglycine.
B. Presumed metabolic origin of ethyl-branched acylglycines compounds containing 10 carbons.
C. EIC of 10-carbon acylglycine isomers in urine and corresponding M+3 isotopologues, after feeding mice with labeled ethylmalonate.
D. Presumed metabolic origin of ethyl-branched acylglycines compounds after feeding the mice with labeled ethylmalonate.
E-G. EIC of 10-carbon (E) keto-acylglycine isomers, (F) hydroxy-acylglycine isomers, (G) enoyl-acylglycine isomers.
Presumed ethyl-branched species are filled in black. Numbering of peaks on the chromatograms correspond to the numbering in Table 1.

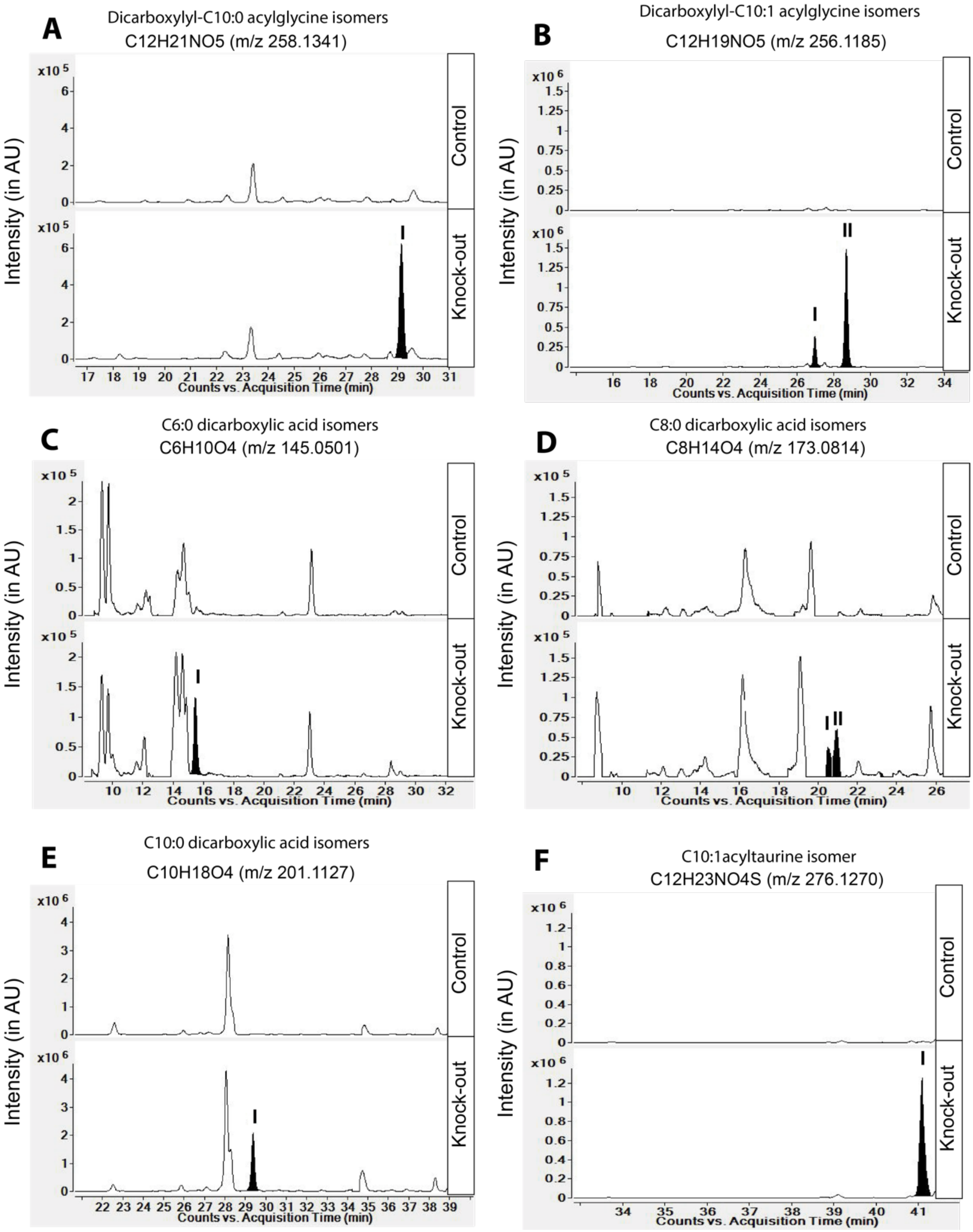

FIGURE S6. Additional extracted ion chromatograms of ethyl-branched acyl derivatives found in urine.
A. Dicarboxylyl-C10:0 acylglycine isomers.
B. Dicarboxylyl-C10 :1 acylglycine isomers.
C. C6:0 dicarboxylic acid isomers.
D. C8:0 dicarboxylic acid isomers.
E. C10:0 dicarboxylic acid isomers.
F. C10:1 acyltaurine isomer.
Putative ethyl-branched species are filled in black. Numbering of peaks on the chromatograms correspond the numbering in Table 1.

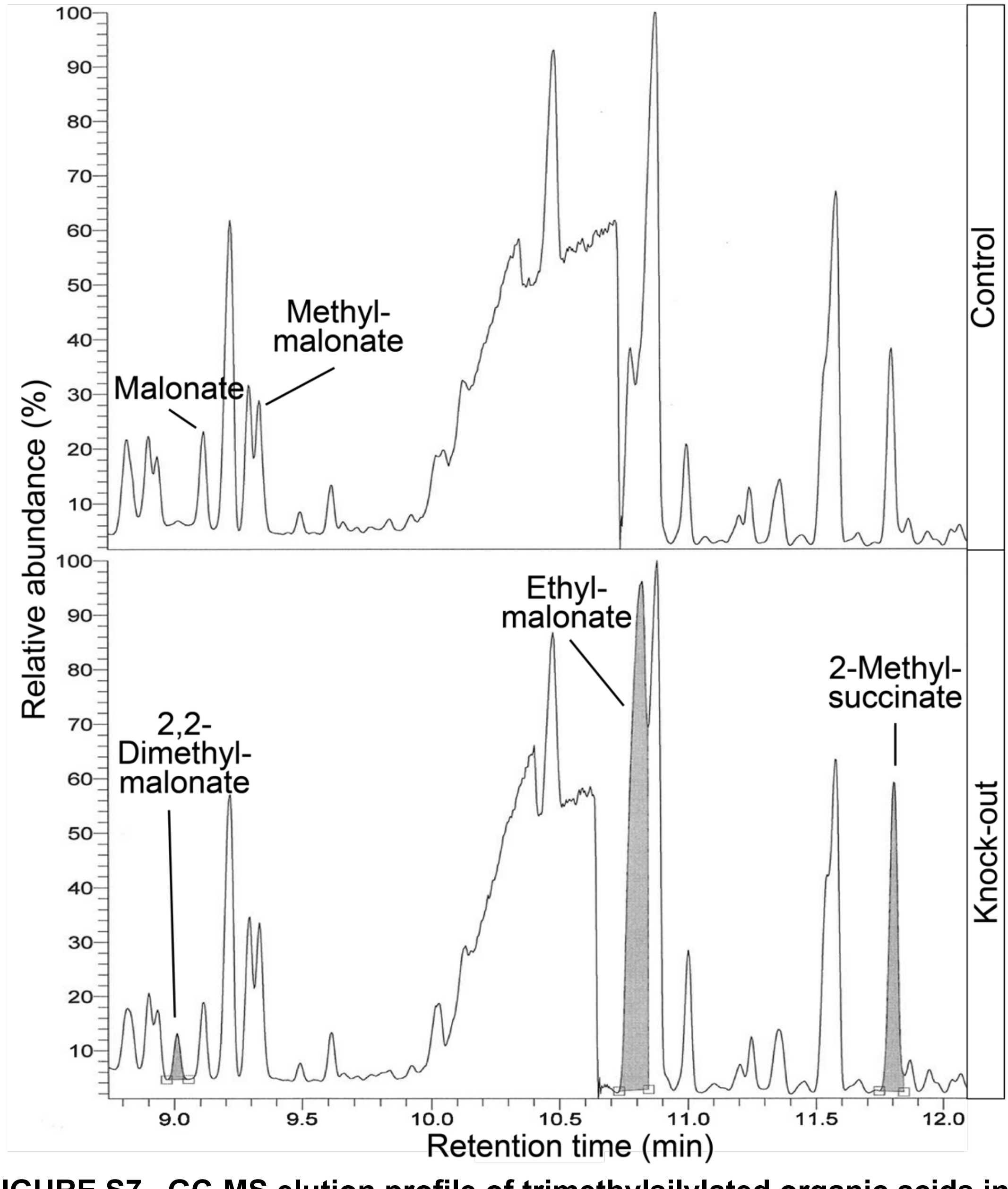

FIGURE S7. GC-MS elution profile of trimethylsilylated organic acids in urines. 2,2-dimethylmalonate, ethylmalonate and methylsuccinate are well resolved using this method.
